# A G-quadruplex-binding platinum complex induces cancer mitochondrial dysfunction through dual-targeting mitochondrial and nuclear G4 enriched genome

**DOI:** 10.1186/s12929-024-01041-6

**Published:** 2024-05-13

**Authors:** Keli Kuang, Chunyan Li, Fatlinda Maksut, Deepanjan Ghosh, Robin Vinck, Maolin Wang, Joël Poupon, Run Xiang, Wen Li, Fei Li, Zhu Wang, Junrong Du, Marie-Paule Teulade-Fichou, Gilles Gasser, Sophie Bombard, Tao Jia

**Affiliations:** 1 Key Laboratory of Drug-Targeting and Drug Delivery System of the Education Ministry and Sichuan Province, Sichuan Engineering Laboratory for Plant-Sourced Drug and Sichuan Research Center for Drug Precision Industrial Technology, West China School of Pharmacy, Sichuan University, 610041 Chengdu, China; 2 CNRS-UMR9187, INSERM U1196, PSL-Research University, 91405 Orsay, France; 3 CNRS-UMR9187, INSERM U1196, Université Paris Saclay, 91405 Orsay, France; 4 Chimie ParisTech, Institute of Chemistry for Life and Health Sciences, Laboratory for Inorganic Chemical Biology, PSL University, CNRS, F-75005 Paris, France; 5 Hôpital Lariboisière (AP-HP), Laboratoire de Toxicologie Biologique, 2 rue Ambroise Paré, 75475 Paris, France; 6 Department of Thoracic Surgery, Sichuan Clinical Research Center for Cancer, Sichuan Cancer Hospital & Institute, Sichuan Cancer Center, Affiliated Cancer Hospital of University of Electronic Science and Technology of China, Chengdu, China; 7 Department of Medical Oncology, Cancer Center, West China Hospital, Sichuan University, Chengdu, China

**Keywords:** G4, Mitochondrial genome, Mito-Nuclear interactions, ROS, Platinum complex, Chemotherapy

## Abstract

**Background:**

G-quadruplex DNA (G4) is a non-canonical structure forming in guanine-rich regions, which play a vital role in cancer biology and are now being acknowledged in both nuclear and mitochondrial (mt) genome. However, the impact of G4-based targeted therapy on both nuclear and mt genome, affecting mt function and its underlying mechanisms remain largely unexplored.

**Methods:**

The mechanisms of action and therapeutic effects of a G4-binding platinum(II) complex, Pt-ttpy, on mitochondria were conducted through a comprehensive approaches with in vitro and in vivo models, including ICP-MS for platinum measurement, PCR-based genetic analysis, western blotting (WB), confocal microscope for mt morphology study, extracellular flux analyzer, JC1 and Annexin V apoptosis assay, flow cytometry and high content microscope screening with single-cell quantification of both ROS and mt specific ROS, as well as click-chemistry for IF study of mt translation. Decipher Pt-ttpy effects on nuclear-encoded mt related genes expression were undertaken via RNA-seq, Chip-seq and CUT-RUN assays.

**Results:**

Pt-ttpy, shows a highest accumulation in the mitochondria of A2780 cancer cells as compared with two other platinum(II) complexes with no/weak G4-binding properties, Pt-tpy and cisplatin. Pt-ttpy induces mtDNA deletion, copy reduction and transcription inhibition, hindering mt protein translation. Functional analysis reveals potent mt dysfunction without reactive oxygen species (ROS) induction. Mechanistic study provided first evidence that most of mt ribosome genes are highly enriched in G4 structures in their promoter regions, notably, Pt-ttpy impairs most nuclear-encoded mt ribosome genes’ transcription through dampening the recruiting of transcription initiation and elongation factors of NELFB and TAF1 to their promoter with G4-enriched sequences. In vivo studies show Pt-ttpy’s efficient anti-tumor effects, disrupting mt genome function with fewer side effects than cisplatin.

**Conclusion:**

This study underscores Pt-ttpy as a G4-binding platinum(II) complex, effectively targeting cancer mitochondria through dual action on mt and nuclear G4-enriched genomes without inducing ROS, offering promise for safer and effective platinum-based G4-targeted cancer therapy.

**Graphical Abstract:**

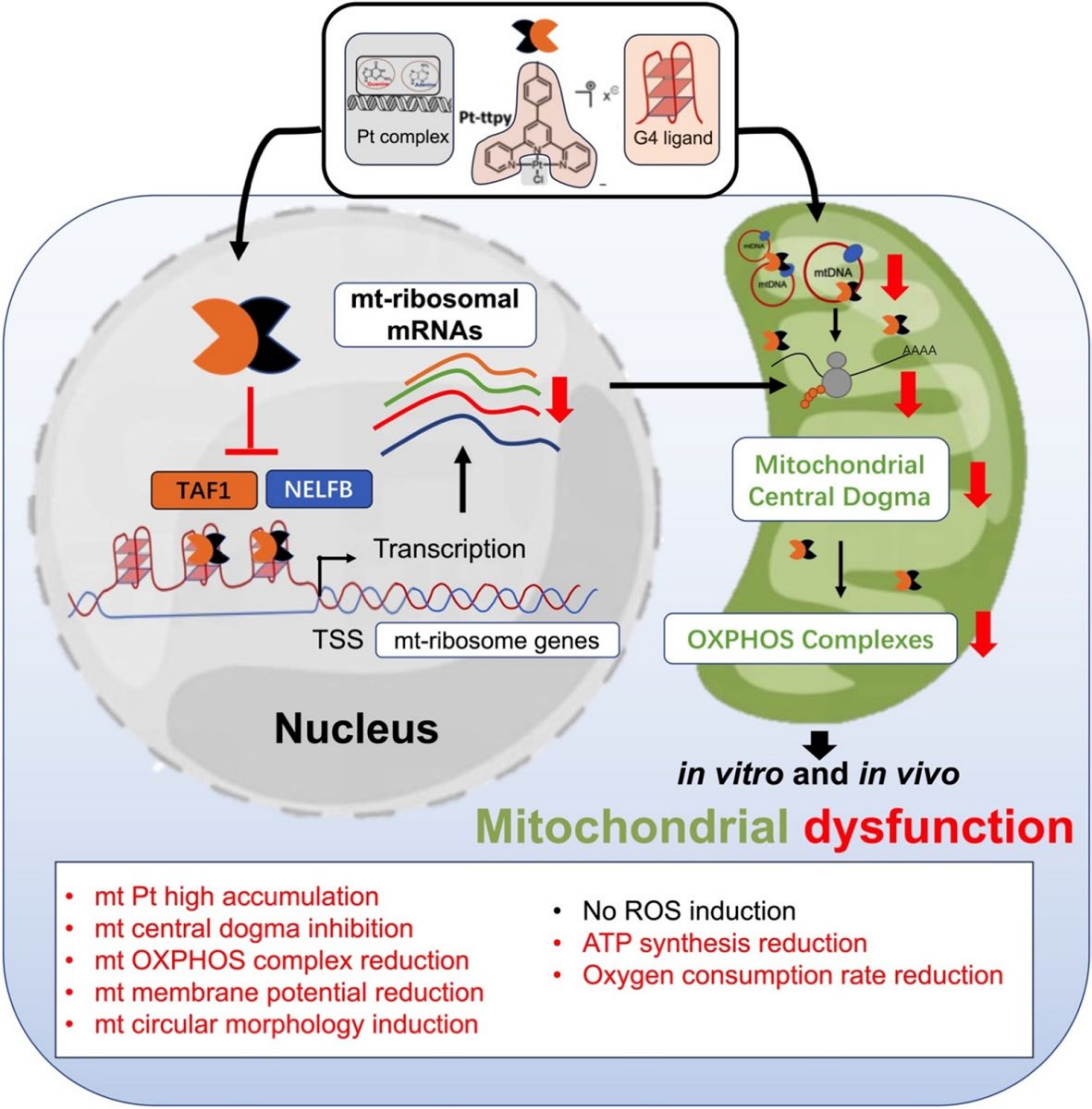

**Supplementary Information:**

The online version contains supplementary material available at 10.1186/s12929-024-01041-6.

## Introduction

G-quadruplex(G4) structures are non-canonical unique secondary four-stranded nucleic acids structures folded in guanine-rich repetitive DNA or RNA sequences. They consist in the stacking of guanine tetramers linked together by Hoogsteen hydrogen bonding, that are stabilized via π-π interactions and monovalent cations [1, 2]. The human genome contains about 350,000-600,000 potential G4 sequences by computer prediction [3, 4]. Analysis of in vitro polymerase stop assays revealed approximately 700,000 G4 structures (5), however, when employing the G4 ChIP-seq assay, the frequency of G4s in human chromatin decreases significantly to be roughly 10,000 [5]. G4 structures have been extensively studied in nuclear DNA, mainly clustered in key regions of the genome: telomeres, gene promoters and DNA replication start points [6, 7]. Recent emerging evidence emphasized the G4 presence in mitochondrial DNA (mt DNA) as well [8–10]. Prediction by the G4 Hunter algorithm revealed that the complete genome of a mitochondrion (16.6 kb) has approximately 96 G4s [4]. Given the regulatory potential of G4 structures in mitochondrial processes and their involvement in cancer, targeting G4 structures would have therapeutic implications [11].

Mitochondria are specialized organelles that are at the heart of energy production (ATP) through the oxidative phosphorylation (OXPHOS) pathways and serves as centers of cellular signaling and apoptosis. They possess their own genome that codes notably for RNAs that encode 13 of the protein subunits of OXPHOS complex, the other mitochondrial proteins being encoded by nuclear genes. Mt DNA can be replicated independently of nuclear DNA and G4 structures in mt DNA are closely related to its own replication and transcription [1, 12]. Small molecules that can stabilize G4 structures have been extensively explored as potential therapeutic agents for cancer [13–19]. Some evidence indicated that, in addition to target G4 structures in genomic DNA and RNA, they could also target G4 structures in mt DNA. Notably the G4-ligand, RHPS4, interferes with mitochondrial function through perturbation of mitochondrial genome replication, transcription processivity, and respiratory function in mouse embryonic fibroblast cells [20]. G4 structures are present in both nuclear and mt genome, hence, to explore the potential of G4 structures in the regulation of mitochondrial function for anti-cancer, investigation at both nuclear and mt genome level is warranted, especially when exploring the use of G4 ligands.

**Scheme 1:**
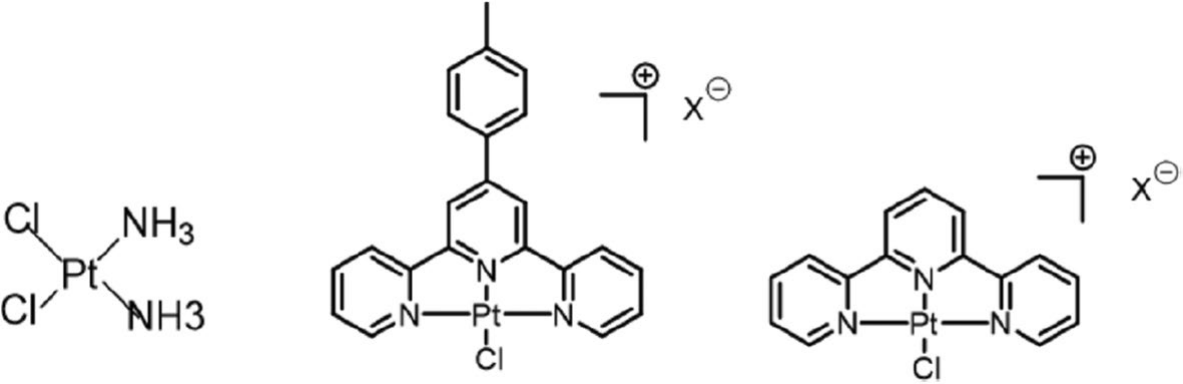
Chemical structures of cisplatin (CisPt), Pt-ttpy and its terpyridine analogue Pt-tpy

We have previously reported on the tolyl-terpyridin-platinum complex (Pt-ttpy) (Scheme 1) that stabilizes G4s in vitro preferentially to duplex DNA through stacking to external G-tetrads [21, 22]. This compound is also able to efficiently trap G4s covalently by direct coordination to loop bases [23, 24]. Our previous cellular and molecular mechanism studies indicate that Pt-ttpy binds covalently to telomeric DNA *in cellulo* [25], inducing chromosome loss and ultrafine bridges formation, resulting in telomeric DNA damage and telomere deprotection [26, 27], by inducing DNA damage preferentially at G- and A-rich regions, displaying potent anti-tumor activity.

In this study, we first demonstrated that Pt-ttpy exhibits strong disruption in mitochondrial function which is unrelated to the induction of reactive oxygen species (ROS). mt dysfunction was induced by G4 targeting in both nuclear and likely in mitochondrial genome as an alternative mechanism underlying the anti-tumor activity of Pt-ttpy by in vitro and in vivo studies. Secondly, we showed that Pt-ttpy displays effective anti-cancer benefits with relative improved safety, which can be attributed to its induction of mt dysfunction without production of ROS, thus reducing treatment-related side effects commonly associated with platinum complexes, e.g. cisplatin. Lastly, we provided first evidence that most mt ribosome genes that are highly enriched in G4 structures in their promoter regions, are the targets of Pt-ttpy. The latter inhibits their gene expression through dampening the recruitment of TAF1 and NELFB to their corresponding promoters in nuclear DNA that ultimately leads to induction of mt dysfunction. These findings lead to the promise for developing G4-binding platinum-based compounds with improved safety profiles as well as effective anti-cancer benefits.

## Materials and methods

### Cell culture

Ovarian cancer cell line A2780 (catalog no. CTCC-003-0011, Meisen CTCC), Cervical cancer cell line Hela (catalog no. CTCC-001-0006, Meisen CTCC), non-small cell lung cancer (NCLC) cell line H2170 (catalog no. CTCC-400-0050, Meisen CTCC) and Mouse primary Lung micro-endothelial cells were purchased from Zhejiang Meisen Cell Technology Co., Ltd. Oral squamous cell carcinoma cell line Cal27 is a kind gift from the lab of Prof. Qin He from West China school of Pharmacy, and NCLC cell lines H520 and SK-MES-1 are kind gifts from the lab of Prof. Zhoufeng Wang from West China hospital. Normal colon epithelial cells HcoEpic and NCM460 are kind gifts from the lab of Prof. Yinglan Zhao from State Key Laboratory of Biotherapy, Sichuan University. Human primary lung fibroblast cells were sorted by CD106 antibody (#130-122-339, Miltenyi Biotec) with the kit of Dynabeads™ FlowComp™ Flexi (#11061D, ThermoFisher). Human cancer cells A2780, H2170, H520 and human primary fibroblast cells were cultured in complete RPMI 1640 medium supplemented with 10% fetal bovine serum (FBS, catalog no. Z7185FBS-500, ZETA life) and 100 U/mL penicillin + 100 ug/mL streptomycin (catalog no. Gibco-15,140,122, ThermoFisher, Gibco). Cancer cells Hela, and Cal27 were cultured with DMEM medium with 10%FBS with penicillin and streptomycin. Cancer cells SK-MES-1 were cultured with MEM-α medium with 10%FBS with penicillin and streptomycin. Primary mouse lung micro-endothelial cells were cultured with Lonza EGM-2 MV microvascular endothelial cells growth medium-2 Bulletkit (#CC-3202). Cells were incubated under a 5% CO2 humidified incubator at 37℃. When it reached 80-90% fusion, cells were digested with 0.25% trypsin/0.91 mM EDTA (catalog no. Gibco 2,520,072, ThermoFisher), then collected for indicated experiments.

### Platinum complexes

Cisplatin (CisPt) was provided from MCE. MedChemExpress (catalog no. HY-17,394). Pt-ttpy (tolylterpyridine platinum complex) and Pt-tpy (terpyridine platinum complex) were synthesized following the procedure already described [22] (Scheme 1). Pt-ttpy was also provided from Merck Sigma (catalog no. SML2556). Aqueous solutions of 1 mM cisplatin, of 1 mM Pt-tpy, and 6 mM DMSO (catalog no. D2650 10 mL, Merck, Sigma Aldrich) solutions of Pt-ttpy were prepared and conserved at − 20 °C. Diluted solutions of each molecule were freshly prepared. The drugs were used at their iso-effect concentrations that inhibit 80% (IC_80_ concentrations) cell proliferation after 96 h that are 0.6 µM, 5.5 µM and 7.5 µM, for cisplatin, Pt-ttpy and Pt-tpy, respectively, unless indicated otherwise.

### Platinum measurement

The platinum cellular uptake was quantified by ICP-MS (Inductively Coupled Plasma Mass Spectrometry, NexION® 2000, Perkin Elmer, Courtaboeuf, France) on cellular pellets (5 × 10^6^ cells), DNA extracts as previously described [27], and on isolated mitochondria. A2780 cells were treated with the IC_80_ concentration of cisplatin, Pt-tppy and Pt-tpy for 96 h. DNA (quantified by nanodrop) was extracted from cell pellets using the DNeasy Blood & Tissue Kit (Qiagen) and mitochondria were isolated using the Mitochondria Isolation Kit for Cultured cell pellets (2 × 10^7^ cells) (Thermo Scientific). Prior to ICP-MS, the samples were digested with pure nitric acid (PlasmaPURE® Plus HNO_3_ 67–69%, SCP Science, Courtaboeuf, France) at 95 °C for cell pellets, and HNO_3_ 0.1 M for DNA and mitochondria. The Pt content was determined following a dose response curve established from known concentrations of platinum. The amount of platinum was then reported as ng of Pt/5 × 10^6^ cells for pellets, pg Pt/µg DNA or ng of Pt/5 × 10^6^ cells for mitochondria.

### Measurement of mitochondrial respiration

A2780 cells were seeded in a Seahorse XF96 96-well cell culture plate (Agilent) (8,000 cells/well, 80 µL of RPMI medium completed with 10% FBS and penicillin/streptomycin). The plate was incubated for 1 h at r.t. and then at 37 °C, 5% CO_2_ overnight. Cells were then treated with compounds dilutions (10 µM) and incubated for an additional 24 h at 37 °C, 5% CO_2_. The seahorse Mitostress test was then performed in accordance with the manufacturer instructions using inhibitors solution at the following final concentrations: [oligomycin] = 1.5 µM, [FCCP] = 0.5 µM, [Rotenone] = [Antimycin A] = 0.5 µM. Following the assay, the medium was carefully removed, and cells were fixed with 100 µL of 4% PFA in PBS for 10 min at room temperature. Cells were then washed twice with PBS and incubated with 100 µL of ca. 3 µM Hoechst 33,342 (NucBlue™) for 10 min at r.t. Cells were washed twice with PBS and directly imaged with a Cytation 5 (Agilent) using a 4X objective focused on the center of the well and a DAPI imaging cube. Raw assay data were normalized using the cell coverage in each well image using the Gen5 software.

### Total ROS or mitochondrial ROS detection by FACS

For total ROS production detection, A2780 cells are cultured with the initial concentration of 0.2 × 10^5^ cells/ml in a 6 well plate at their IC_80_ concentration (or 10 µM). After 96 h (or 24 h) treatment, CellROX Deep Red (Molecular Probes) was added at the final concentration of 500-1000nM to the cells and incubated at 37 °C. After washing with PBS, analyze was performed using the flow cytometry and detection at 635 nm excitation for the CellROX Deep Red reagent (Invitrogen).

For Mitochondria ROS detection, it is based on the modified protocol from MitoSOX (M36008, Invitrogen)-based FACS method [28]. Cells are cultured with the initial concentration of 0.5 × 10^6^ cells/ml in a 6 well plate with the complexes Pt-ttpy, Pt-tpy and cisplatin at the concentration of 10 µM. After 24 h treatment, cells were washed with pre-warm PBS in 6-well plate for 1 time. After, 1 µM Mito-sox was added in each well and incubated for 30 min at 37 °C. Wash cells thoroughly with pre-warm PBS for another 3 times, followed by trypsin and cells collection. Using loading buffer (2% FBS in PBS) to collect and mix well cells (working volume is 500 µl) and move to BD FACSCanto studies.

### Total ROS and mitochondrial specific ROS simultaneously detection by high content microscope screening followed by single cell quantification

Investigating simultaneously the total ROS (ROS Assay Kit-Highly sensitive DCFH-DA, #R252, DOJINDO) and Mitochondria ROS (mtSOX Deep Red-Mitochondrial Superoxide detection #MT14, DOJINDO) induction post Pt-ttpy or cisplatin treatments was performed following manufacturer’s protocol. Briefly, indicated cells were treated with cisplatin and Pt-ttpy for 1 day at the con. of 10 µM for cancer cell lines or 1µM for primary cells. After, the living cells was incubated simultaneously with different dye for 30 min at 37 °C. Then, the images were collected with ECLIPSE Ni-E (Nikon) microscope with highly sensitive camera, FITC channel for the detection of total ROS (ex: 488 nm), Red channel for the detection of mt-ROS (ex:555 nm). Single cell fluorescence intensity was unbiased quantified by Image J using in-house developed Macros, at least 50 cells were quantified for each group. And the quantification results were statistically analysed using GraphPad Prism 9.0.

### Fluorescent quantitative PCR and fluorescent quantitative RT-PCR

SYBR probes (POWRUP SYBR MASTER MIX, catalog no. A25742, applied biosystems by Thermo Fisher Scientific) were used in a 25 µl system. Reaction conditions were following the manufacturer’s protocol.

For in vitro samples, total RNA from A2780 cells untreated and treated by the various platinum complexes at their IC_80_ concentrations for 96 h was extracted using the RNA simple Total RNA kit (catalogue no. DP419, TIANGEN), and then taken 1 µg after quantification for reverse transcription. After removal of residual DNA using DNase I, RNase-free (catalogue no. EN0529, Thermo scientific), RevertAid MM (catalogue no.M1631, Thermo scientific) was added and reversed to cDNA using a PCR instrument (Bio-Red).

For in vivo samples, tumor tissue DNA was extracted using the FastPure®DNA Isolation Mini Kit (catalogue no. DC112-02, Vazyme) and diluted to 10 ng/ml. Total tumor tissue RNA was extracted using the FastPure®Total RNA Isolation Mini Kit (catalogue no.RC112-01, Vazyme) and subsequently reverse transcription was performed as before. Real-time qPCR was carried out using a QuantStudio 3 Real-Time qPCR System (Applied Biosystems). The primers used are shown in the Supplementary Table S1.

### qPCR-based method for quantification of mtDNA copy numbers including deleted and non-deleted isoforms

**Fig. 1:**
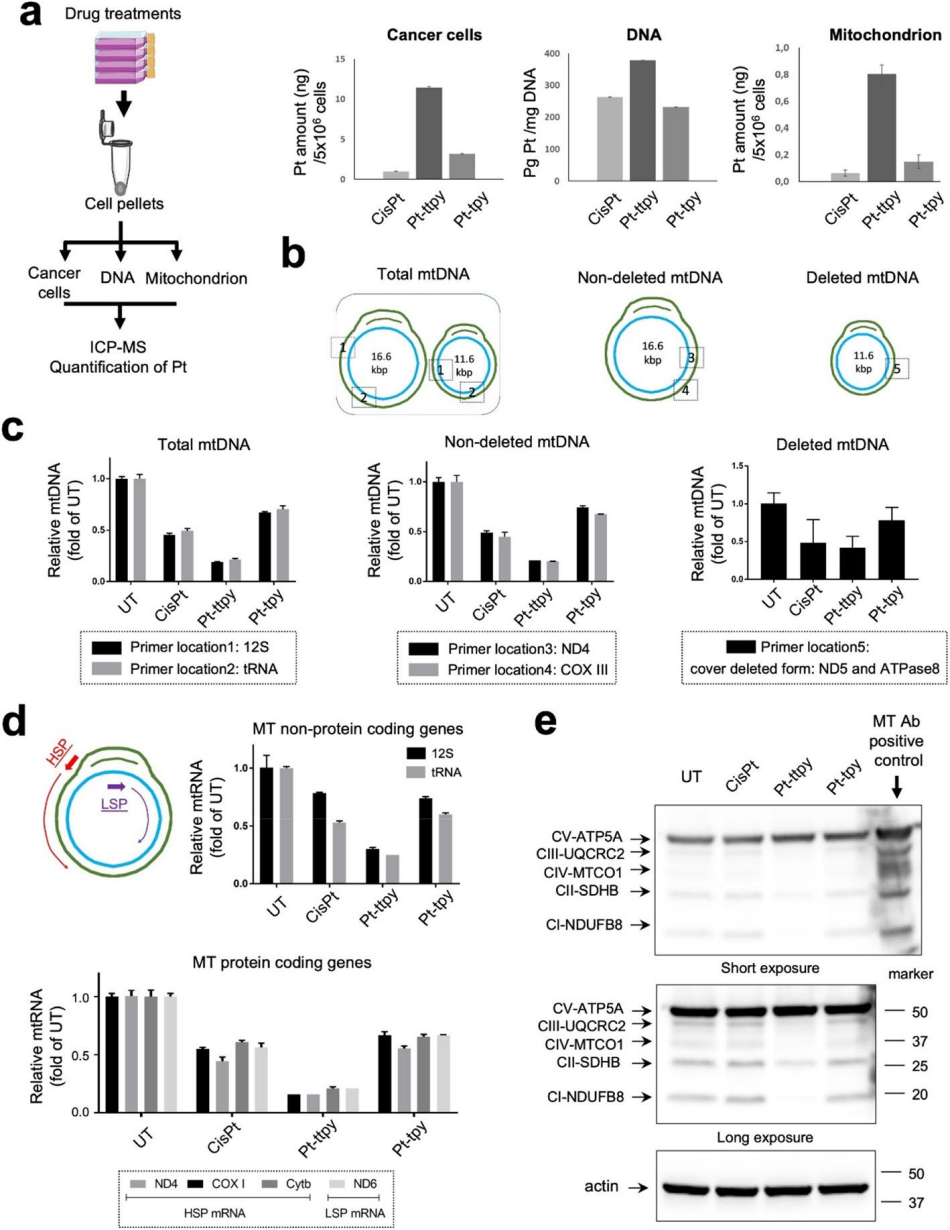
Impact of different platinum (Pt) complexes (cisplatin, Pt-ttpy and Pt-tpy) on cellular uptake and distribution with the potential toxicity to mitochondrial genome at their IC80 concentration in A2780 treated cells. **a** Schematic illustration of platinum quantification flow in cell pellets, genomic DNA and mitochondria is presented in the left, comparative quantification of Pt amount(ng)/5 × 10^6^ cells for cisplatin, Pt-ttpy and Pt-tpy was performed in cell pellet, extracted genomic DNA and isolated mitochondria, respectively after 96 hours treatment. Data represents three independent experiments with the mean ± SEM. **b** A sketch of describing different primers’ position in non-deleted mt-DNA, deleted mt-DNA and total mt-DNA is presented, that is used for qPCR analysis as presented in figure c. **c** qPCR quantification of different mt-DNA copy numbers under different Pt complexes treatments after 96 hours treatment, data is presented as relative fold changes of mtDNA copy numbers for different Pt complexes’ treatment groups compared to the untreated (UT) group. Data represents three independent experiments with the mean ± SEM. **d** RT-qPCR quantification of different mt-RNA levels, including both mt non-protein coding genes and its protein coding genes in response to different Pt complexes’ 96h treatment groups compared to the UT group. Data represents three independent experiments with the mean ± SEM. **e** Western blot study of different mt OXPHOS complex proteins in the 96 hours treatment of different Pt complexes. Also shown is a blot of actin as a loading control. The corresponding quantification data of different mt OXPHOS complex protein levels is presented in the supplementary Fig. 2. Data represents two independent experiments

Investigating the relative changes of mtDNA copy numbers is based on qPCR method. Total DNA for indicated in vitro cell samples untreated and treated by the various platinum complexes at their IC_80_ concentrations for 96 h or in vivo tumor samples were extracted using DNA Blood and Tissue Kit (Qiagen, Germany). DNA quantity was determined by NanoDrop (Thermo Fisher). The DNA showed a high purity (A260/ A280 > 1.8) and was stored at -20 °C. The primers used for real time amplification were synthesized and HPLC-purified by Eurogentec. Because the most common aberrancy is a 4,977-bp deletion spanning nucleotides 8,483–13,459 of the mitochondrial genome [29], different primers were used for detecting mtDNA deleted (also known as mtDNA^4977^) and non-deleted isoforms, as well as total mtDNA including both isoforms. Their primers’ location are indicated in Fig. 1b. The primers of 12 S, tRNA are used for quantification of total mtDNA, and the primers of ND4 and COX III are used for quantification of non-deleted mtDNA. All the above primers are listed in the Supplementary Table 1. The primers used for deleted mtDNA isoform (mtDNA^4977^) quantification is covering the gene ND5 and ATPase8, and common deletion primer Forward: TTCCTCATCACCCAACTAAAAA, common deletion primer Reverse: TTCGATGATGTGGTCTTTGG. Real-time qPCR was carried out using a QuantStudio 5 real-time PCR system by conventional settings (Applied Biosystems).

### RNA sequence and analysis

A2780 cells were plated in 10 cm dishes and divided into three groups: UT, Pt-ttpy and cisplatin. The cell seed densities for each group were as follows: 0.5 × 10^6^ cells per dish for UT, 1.5 × 10^6^ and 1.5 × 10^6^ cells per dish for both Pt-ttpy and cisplatin. After a two-hours incubation, the corresponding drugs were added to each group at their IC_80_ concentration for 96-hours treatment period. At the end of the incubation period, the cells were washed twice with Hank’s Balanced Salt Solution (HBSS, catalog no. C14175500BT, ThermoFisher, Gibco), subsequently, they were treated with the cell lysis solution TRIzoL (catalogue no. 15,596,026, ThermoFisher, invitrogen) at room temperature for 5 min. Afterward, the cells were gently scraped off with a cell scraper and collected in centrifuge tubes, remained at room temperature for an additional 5 min. Finally, the cell samples were snap-frozen in liquid nitrogen and stored in an ultra-low temperature refrigerator, which would be used for RNA extraction just before conducting RNA sequence. Three distinct and independent samples were collected for each group.

RNA transcriptomics sequencing was conducted by Biomarker Technologies on each set of three parallel samples. After successfully passing the library quality check, pooling was performed according to the target downstream data volume and sequencing was carried out using the Illumina platform. Clean data was filtered for sequence alignment with the reference human genome, and mapped data was obtained for library quality assessment such as insert length testing and randomness testing. Structural-level analysis such as variable splicing analysis, novel gene discovery and gene structure optimization were also performed. Differential gene expression analysis was conducted to identify differences in gene expression among different samples or sample groups. The data was graphically by R for MacOSX to generate the figures included in the manuscript.

To identify genes related to nuclear-encoded mitochondrial proteins that were down-regulated specifically by Pt-ttpy treatment, we firstly identified the genes exhibiting down-regulation in either Pt-ttpy or Cispt treatment groups, as compared to the untreated (UT) group (with criteria FDR < 0.05, FC > 1.2). It leads to the creation of two distinct gene cohorts. Subsequently, we intersected these two gene cohorts with a public human mitochondrial gene cohort (MitoCarta 3.0, https://personal.broadinstitute.org/scalvo/MitoCarta3.0/human.mitocarta3.0.html) [30]. After, we employed Venn diagrams, as depicted in Fig. 5b, to generate a set of genes (106) that were specifically down-regulated in the Pt-ttpy group, not in the cisplatin group. These mitochondria-related genes are supposed to be specially downregulated by the unique G4-binding property of Pt-ttpy.

Conducting a differential gene expression analysis by transcriptome sequencing data obtained from A2780 cells subjected to different treatments with CisPt and Pt-ttpy involved the utilization of the DESeq2 package. Subsequently, heatmaps representing differentially expressed genes (DEGs) were created within RStudio (version 4.3.2). Furthermore, gene ontology (GO) and KEGG pathway analyses were executed on the BMKCloud platform (http://www.biocloud.net/) to annotate relevant genes and elucidate enriched biological processes and signalling pathways.

### CUT&RUN-qPCR

Experimental reagents were used with Vazyme’s Hyperactive pG-MNase CUT&RUN Assay Kit for PCR/qPCR (catalogue no. HD101, Vazyme). A2780 cells were plated in 10 cm dishes in DMSO and Pt-ttpy groups at a cell density of 1 × 10^6^ and 3 × 10^6^. After two hours of seeding, Pt-ttpy at IC_80_ concentration was added in indicated dishes. And a corresponding volume of DMSO (< 1%) was added to the DMSO group as control and incubated at 37 °C for 96 h. Cells were collected by trypsin digestion and dispensed into 0.5 × 10^6^/ tubes. Subsequent steps were performed according to the experimental protocol [33]. Specific antibodies are used to bind to transcription complexes and pull-down specific fragments of DNA sequences by enzymatic cleavage and purification: TAF1 (TAF1 Rabbit mAb catalogue no.#12781S, D6J8B, CST) and NELFB (COBRA1 Rabbit mAb catalogue no.#14894S, D6K9A, CST) antibodies. After quantification of the pulled-down DNA sequences, primers, and probe SYBR were added for qPCR. Five pairs of primers were designed in the 1.5 Kb region around the TSS of the *MPV17L2* and *MRPS18C* genes to examine the distribution of different transcription factors and the effect of the drug Pt-ttpy on them. The samples subjected to qPCR expression analysis using SYBR Green probe by PowerUp SYBR Green Master Mix (catalog no. A25742, Applied Biosystems from Thermo Fisher Scientific). The PCR amplification was performed on QuantStudio 3 Real-Time PCR System (Applied Biosystems) with the conventional setting parameters, 40 cycles at 95 °C for 15 s, 60 °C for 1 min. The Mean threshold cycles were determined from three technical repeats using the comparative CT methodology. To standardize expression levels, they were normalized to that of actin.

### Click-chemistry for IF study of MT translation with single cell quantification

A2780 or Hela cells were inoculated on 14 mm coverslips (catalogue no.WHB-24-cs, WHB) in 24-well plates, walled for 4 h and then treated with the Pt-ttpy (IC_80_: 5.5 µM) and corresponding DMSO for 96 h. The medium was gently refreshed by L-Methionine-free 1640 medium (catalogue no.CTCC-002-148,Meisen CTCC) and each well was treated with 100 µg/ml Cycloheximide (CHI, catalogue no.HY-12,320, MedChemExpress) and incubated for 30 min at 37 °C to stop protein translation in the cytoplasm ; In addition to this positive control, 80 µg/ml of Chloramphenicol (catalogue no.HY-B0239, MedChemExpress) was added and incubated for 30 min at 37 °C to stop mitochondrial protein translation; 500µM of methionine analogue-homoacetylglycine HPG (catalogue no.HY-140,345 A, MedChemExpress) was added to each well and incubated for 60 min at 37 °C to insert it into the nascent protein peptide chain. Before fixation, cells were permeabilized in pre-chilled buffer A (10 mM HEPES; 10 mM NaCl; 5 mM MgCl_2_ ; 300 mM sucrose) containing 0.015% digitonin (catalogue no.HY-N4000, MedChemExpress) for two minutes, followed by 15 s reaction in buffer A without digitonin; 4% PFA ( catalogue no.BL539A, Biosharp) fixed cells for 10 min, washed that in PBS and permeabilized that in 0.1% Triton X-100 (catalogue no.9002-93-1, Solarbio) for 20 min; 3% BSA (catalogue no.9048-46-8, Merck Sigma) was used for blocking for another 30 min and the cells were treated with 20 µM of Alexa Fluor® 488 (labeled to azide, catalogue no.A10266, ThermoFisher, Invitrogen) that had been diluted to the antibody reaction solution (100 mM Tris, 100 mM ascorbicacid, 1 mM CuSO_4_) for the click reaction in 15 min at room temperature [34]; after washing with PBS, 1 µg/ml of DAPI (catalogue no.28718-90-3, MedChemExpress) was added at room temperature for another 5 min. After washing with PBS, slides were sealed to air-dry in hood. Images were collected with ECLIPSE Ni-E (Nikon) microscope using oil with the 40x objective.

Single cell fluorescence intensity was unbiased quantified by Image J (version 1.54f) using in-house developed Macros, at least 200 cells were quantified for each group. And the quantification results were statistically analysed using GraphPad Prism 9.0.

### Immunofluorescence (IF) study of mitochondrial morphology and quantification of mitochondria by FACS (TOMM20 labelling)

For IF studies by TOMM20 labeling, A2780 cells were plated firstly on 8-well labteks (Thermo fish scientific). Cells were treated for 96 h at their respective IC_80_ concentrations (see main text). After treatment, cells were washed with PBS, then fixed 10 min in 2% Paraformaldehyde (PFA). After wash with PBS, cells were permeabilized for 10 min at RT using 0.2% Triton X-100 and washed with PBS. The cells were incubated in blocking buffer (5% goat serum in PBS) for 60 min at RT before being incubated at 4 °C overnight in 1% BSA dissolved in PBS with the primary antibody against TOMM20 (Abcam). On the second day, after three times wash with PBS, the cells were incubated for another 30 min with the Alexa Fluor 555-conjugated secondary antibody (Life Technologies). Nuclei were labeled using DAPI and the cover slides were mounted with VectashieldTM. Acquisitions were performed on Leica SP5 confocal microscope by the microscopy platform of the Institut Curie.

The Mitochondrial circularity value analysis is used to quantify the changes of mitochondrial morphology by Image J. According to the using documents of Image J (version 1.54f), the circularity value of 1.0 indicates a perfect circle. And the circularity value approaches 0, it indicates an increasingly elongated shape.

For FACS studies by TOMM20 labelling, A2780 cells were plated firstly on 10 cm dishes (Thermo fish scientific). Cells were treated for 96 h at their respective IC_80_ concentrations (see main text). After treatment, cells were washed with PBS, followed by trypsin to collect cells. Suspend cells at around 2 × 10^6^ in 250 µl washing buffer (PBS + 0.5%BSA) with another 250 µl 4% PFA. Mix well and fix samples for another 10 min at r.t. After permeabilization with 0.2% Triton X-100, cells were stained with TOMM20 antibody (Abcam) for 15 min by gentle rotation at 4 °C, followed by 3 times thoroughly wash with PBS. After, cells were incubated with 1 µl second antibody-PE in dark for another for another 10 min by gentle rotation at 4 °C (working volume is 200 µl). Wash thoroughly cells with 1mL permeabilization buffer for another 2 times, then prepare 400 µl washing buffer to collect and mix well cells and move that for BD FACSCanto studies.

### Tumor xenografts studies

SPF-rated BALB/c nude mouse (6 weeks old) weighting 20–22 g were purchased from Chengdu Dossy Laboratory Animal Company. Before the beginning of the experiment, animals were acclimatized in a temperature-controlled environment for 1 week. The nude mice were housed in individually ventilated cages fed a normal diet and water under artificially controlled environment (temperature 20 ± 2 °C, humidity 50-60%, photoperiod: 12 h light, 12 h dark). Murine experiments were carried out following the guidelines of medical research and new medical technology of Sichuan Cancer Hospital Ethics Committee and performed under study number SCCHEC-02-2023-064. All methods were performed in accordance with Guide for the Care and Use of Laboratory Animals.

A2780 cells in the exponential growth phase were collected and resuspended in 50 µl of RPMI medium per 1 × 10^7^ cells, and the same volume of cell matrix (catalogue no.356,234, Corning® Matrigel® Matrix) was mixed to the cell suspension in ice. Suspended A2780 cells (1 × 10^7^ cells/mouse) were injected subcutaneously on the back next to the right leg in a sterile environment on an ultra-clean table. After injection for 5 days, they were randomly divided into 3 groups according to the size of the tumor volume (V = L^2^×W × π/6) equally, namely DMSO, Pt-ttpy and cisplatin t groups. The nude mice in the three groups were injected intraperitoneally with 400 µl of 1% DMSO, Pt-ttpy (5 mg/kg) and cisplatin (2 mg/kg) once every two days for 21 days. The body weight and tumor volume of each nude mouse were recorded during this time. When dosing was complete, all nude mice were euthanized and the tumors were isolated, rapidly cooled in liquid nitrogen or stored at ultra-low temperature refrigerator for further studies. Major tissues, including the liver, kidney, and heart, were weighted, the tissue index was calculated as the ratio of tissue weight (g) to body weight (g): tissue index % = liver weight (g) / body weight (g) * 100. Then, a preliminary major tissue toxicity study was performed using typical HE staining.

### Western blotting (WB)

For in vitro cell pellets samples preparation, 0.2–0.3 × 10^6^/well A2780 cells was plated in 6-well plate with full medium for 24 h, then full medium was removed, and cells were refreshed with indicated treatments with metallic complexes at their IC_80_ concentration for 24–96 h. After, total proteins were extracted using RIPA 1X buffer (Cell Signaling Technology, CST) supplemented just before use with 1X EDTA-free Protease Inhibitor Cocktail (Roche), 20 mM NaF and 1 mM Na3VO4. Around 20 µg proteins were loaded onto Mini-PROTEAN Precast gels (BioRAD) for further WB procedures.

For in vivo tumor tissue samples preparation for western blotting, proteins were isolated from tumor tissue using a 3-min ultrasonic cycle homogenization (cycle of 15 s sonication, 10 s resting time) in ice, followed by a 30 min more extraction in ice using RIPA (CST) 1X buffer supplemented just before using with protease and phosphate inhibitors (HY-K0022/K0023, MCE® MedChemExpress). Samples were vortexed for 15 s by every 15 min. After centrifugation for 20 min at 14.000 rpm at 4 °C, supernatants were collected, and protein amount was quantified for each group or mice using BCA. The separation of proteins was performed using either Mini-PROTEAN Precast gels (BioRAD) or NuPAGE 4–12% gels (Life Technologies).

After, proteins were transferred to PVDF membranes (catalogue no.ISEQ00010, Immobilon ®-PSQ, MERCK Millipore Ltd.) with 90 V for 90 min by BioRAD wet transfer system or semi-dry transfer method using Trans-Blot Turbo Transfer system (BioRAD) with the settings of 1.3 A-25 V-7 M. Primary antibodies (MTCO1, 1:1000, Abclonal; β-Tubulin, 1:8000, Abclonal; actin, 1:3000, CST; total OXPHOS human WB antibody cocktail, 1;1000, Abcam) were diluted with 5% BSA and incubated overnight at 4 °C, then the membranes were incubated with secondary antibodies (HRP Goat Anti- Rabbit IgG(H + L), 1:5000, Abclonal, or HRP-conjugated Affinipure Goat Anti-Mouse IgG(H + L), 1:5000, proteintech) at room temperature for another 2 h. WB detection was performed by chemiluminescence (BioRAD) with traditional X-ray films (FIJIFILM) or digital CDD imaging (BioRAD or Vilber). The intensity of indicated band was measured by ImageJ (NIH software).

### Statistical analysis

The data were analysed using GraphPad Prism 9.0 software (San Diego, CA). The results were presented as either mean ± SEM or ± SD as indicated, details of regarding the number of experimental replicates and statistical analyses methods were indicated in the figure legends.

## Results

### Pt-ttpy shows significant highest accumulation within mitochondria, accompanied by a pronounced disturbance toward the Mitochondrial genome

Our previous work showed that the G4-binding Pt-ttpy complex and its terpyridine counterpart Pt-tpy as well as cisplatin (CisPt), the two latter having no to low affinity for G4, accumulate in cells and bind to genomic DNA in a time- and dose- dependent manner in ovarian cancer cells A2780 (see Scheme 1 for structures of compounds). At iso-effect concentrations that achieve an 80% inhibition of cell proliferation over a 96-hour treatment period, there is a marked higher accumulation of Pt-ttpy in cells. This increase is observed alongside a similar level of genomic DNA binding efficiency with the occurrence of DNA damage among all the three Pt complexes, indicated in scheme 1 [27]. Since some G4-binding ligands were shown to accumulate in mitochondria (mt) [20] as well as some platinum complexes [35], we hypothesized, due to the high cellular uptake of Pt-ttpy, that mitochondria could be a privileged target of Pt-ttpy. We therefore quantified the distribution of the three platinum complexes at iso-effect doses that are their respective IC_80_ concentration after 96 h treatment in the ovarian cancer cell line A2780 cells using the ICP-MS method, in whole cells, in mitochondria and their fraction bound to genomic DNA. Consistent with our previous findings, Pt-ttpy shows a significant accumulation in cancer cells (11 and 3 times more than cisplatin and Pt-tpy, respectively) with a slightly higher binding to DNA (1.5 times) [27].Notably, within mitochondria, Pt-ttpy exhibited stronger accumulation than both cisplatin and Pt-tpy (19 and 8 times more, respectively, as shown in Fig. 1a), It suggests that Pt-ttpy may induce more dysfunction of mitochondria, as compared with the two other Pt complexes. Next, we studied the three complexes’ effects on mitochondrial genome function including mt DNA copy number reduction, mt DNA deletion, mt DNA lesion by a real time quantitative PCR method [36] (Fig. 1b and c, Supplementary Fig. S1), inhibition of mt RNA transcription (Fig. 1d) and reduction of the protein levels of mt OXPHOS complexes (Fig. 1e and Supplementary Fig. S2) in A2780 cells. The induction of mitochondrial genome dysfunction was also detected in another two cancer cell lines Cal27 (Oral squamous cell carcinoma) and H2170 (Lung squamous cell carcinoma) indicated by Supplementary Fig. S3. Collectively, these results revealed that Pt-ttpy, due to its higher cellular accumulation and likely to its G4-binding property, shows a high tendency for accumulating in mitochondria with a strong disruption to mitochondrial functions from mt gene replication to its associated protein expression.

**Fig. 2:**
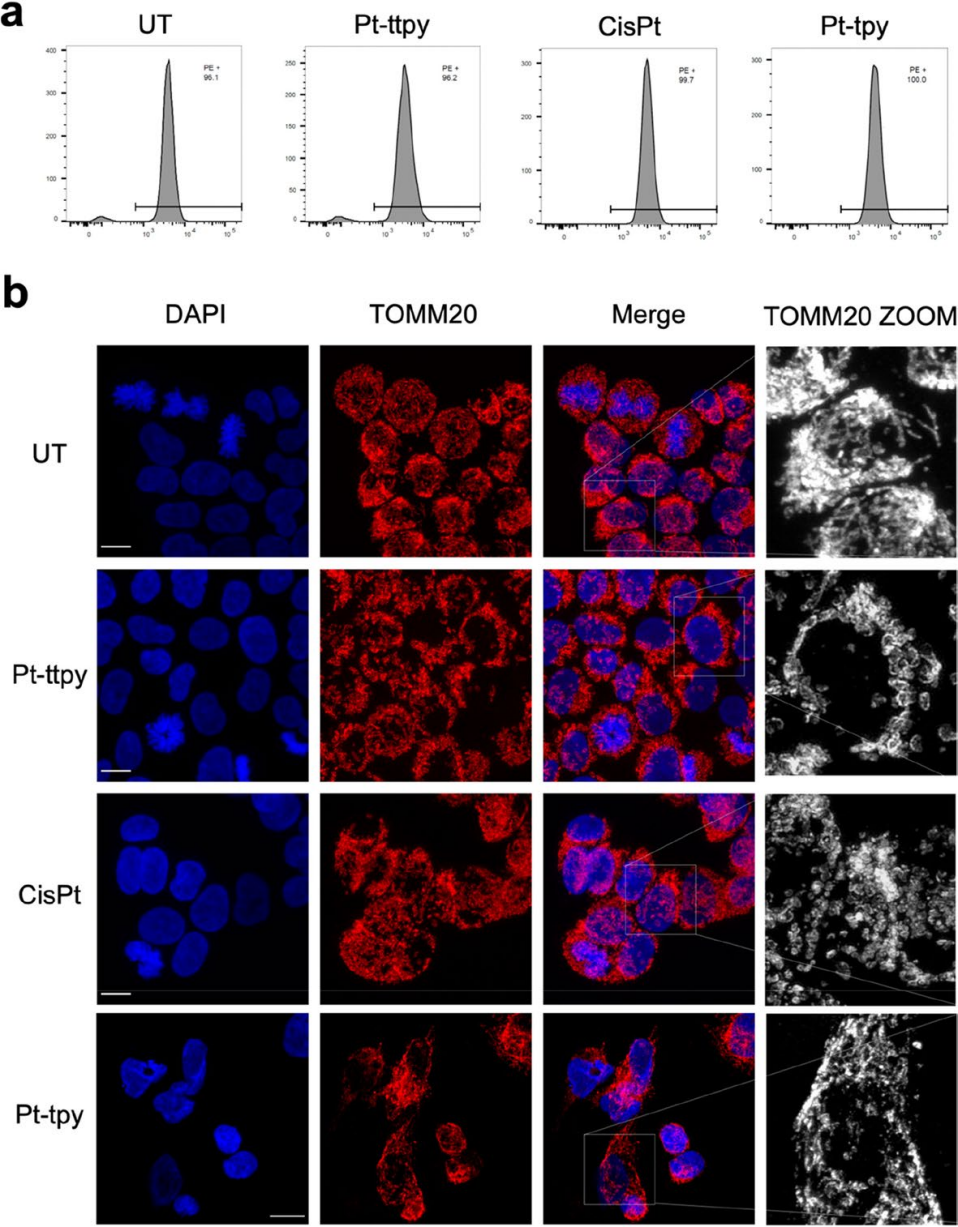
Impact of three platinum (Pt) complexes (cisplatin, Pt-ttpy and Pt-tpy) in mitochondrial number and its morphology of A2780 treated cells. **a** Flow cytometry analysis of mitochondrial number changes in the treatment of different Pt complexes by the staining of TOMM20, plotted are the TOMM20 signal distribution in different treatments. The histogram is represented by two independent experiments. **b** Confocal microscope tested the mitochondrial abundance and its morphology changes following the Pt complexes treatments (cisplatin, Pt-ttpy and Pt-tpy) for 96 h. Scale bar: 10 μm. Data represents three independent experiments

### Pt-ttpy induces a potent mt dysfunction, but without ROS induction

**Fig. 3:**
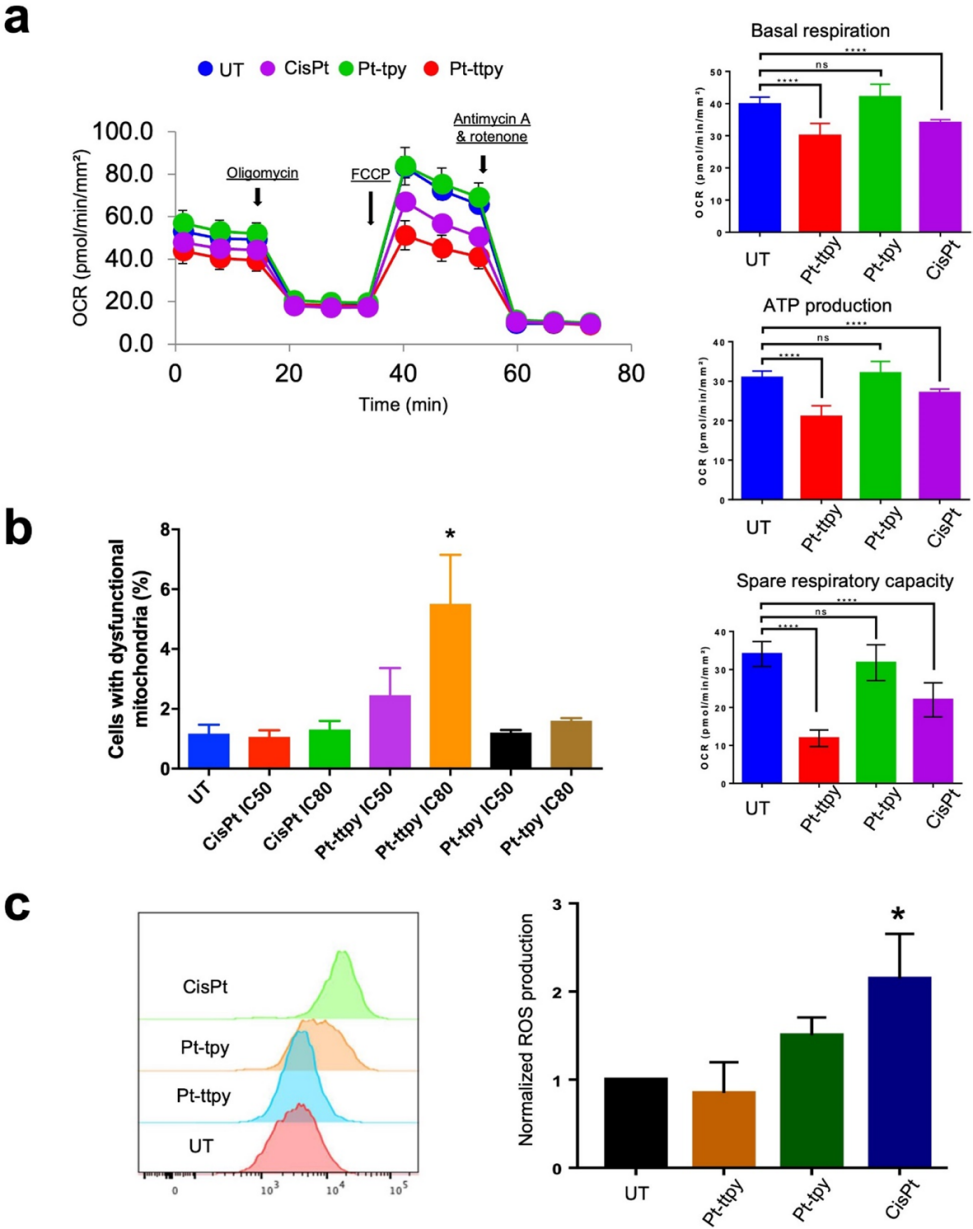
Impact of three platinum (Pt) complexes (cisplatin, Pt-ttpy and Pt-tpy) on mitochondrial homeostasis in A2780 treated cells. **a** Left is presented as the seahorse XF cell mito stress test profile under different Pt complexes treatments (10µM, 24 h treatment) as well as UT group with specific electron transport chain inhibitors: oligomycin (inhibitor of ATP synthase (complex V)), FCCP (uncoupling agent), antimycin-A (complex III inhibitor), and rotenone (complex I inhibitor). Right is plotted as the quantification of basal respiration, ATP production and spare respiratory capacity respectively by different treatments of Pt complexes. **b** Flow cytometry was used to quantify mitochondrial potential changes by the staining of JC1, % Cells with mitochondrial membrane loss (dysfunctional mitochondria) corresponding to the % of cells with JC-1 in its green monomers form after treatment at the respective IC_50_ and IC_80_ concentrations of the complexes. Data represents three independent experiments with the mean ± SEM. **c** Flow cytometry was used to quantify the total ROS production in A2780 cells treated, normalized ROS production is plotted as the mean ± SEM, data represents three independent experiments. P values were calculated toward the UT: *P < 0.05, **P < 0.01, ****P < 0.0001, unpaired t-Student test

To study the consequence of high accumulation of Pt-ttpy in mt with a strong toxicity to mitochondria genome function, we analyzed Pt-ttpy effects on mt function. Interestingly, by FACS and immunofluorescence staining, using TOMM20 antibody (an inner membrane protein of mt), we saw that Pt-ttpy did not induce a significant reduction of the number of mitochondria (Fig. 2a) but a clear mt dysfunctional morphology switch that is also detected in the treatment of cisplatin, but not with Pt-tpy (Fig. 2b and S4). The dysfunctional morphology of mt is consistent with the data collected by real-time mt function monitoring using a Seahorse system (Agilent). mt basal respiration, ATP production as well as spare respiratory capacity were recorded in the presence or absence of the complexes (Fig. 3a). We detected that Pt-ttpy but not Pt-tpy, induced a significant change in cellular respiration (oxygen consumption rate, ATP synthesis and spare respiratory capacity), which suggests mitochondrial dysfunction as one of the modes of Pt-ttpy’s action leading to cancer cell proliferation inhibition. We confirmed that cisplatin disrupts mitochondria respiration [37], but in a less pronounced manner than for Pt-ttpy, in correlation with Mitochondrial genome function impairment. Next, we studied the mitochondrial membrane potential by flow cytometry, one of the hallmarks of mitochondrial damage. The change of mitochondrial membrane potential (Δψ_m_) was detected by JC-1, a well-known probe that accumulates into the mitochondrial membrane matrix space in a manner inversely proportional to Δψ_m_ [38, 39]. Notably, only Pt-ttpy induced a dose-dependent reduction of mt membrane potential (Fig. 3b and Supplementary Fig. S5, which is well correlated to its unique and strong reduction of protein levels of mt OXPHOS complexes, including complexes I, II, III and IV (Fig. 1e and Supplementary Fig. S2). Indeed, there is a strong link between OXPHOS complexes and mitochondrial membrane potential (Δψ_m_), particularly complexes I, III, and IV are intimately involved in establishing and maintaining Δψ_m_ [40]. Additionally, the loss of Δψ_m_ is reported to be as early event in the process of apoptosis [41], and consistently we detected that Pt-ttpy induced relative more early apoptosis signal by the staining of (Annexin V+/7-AAD-) signals (Supplementary Fig. S6).

**Fig. 4:**
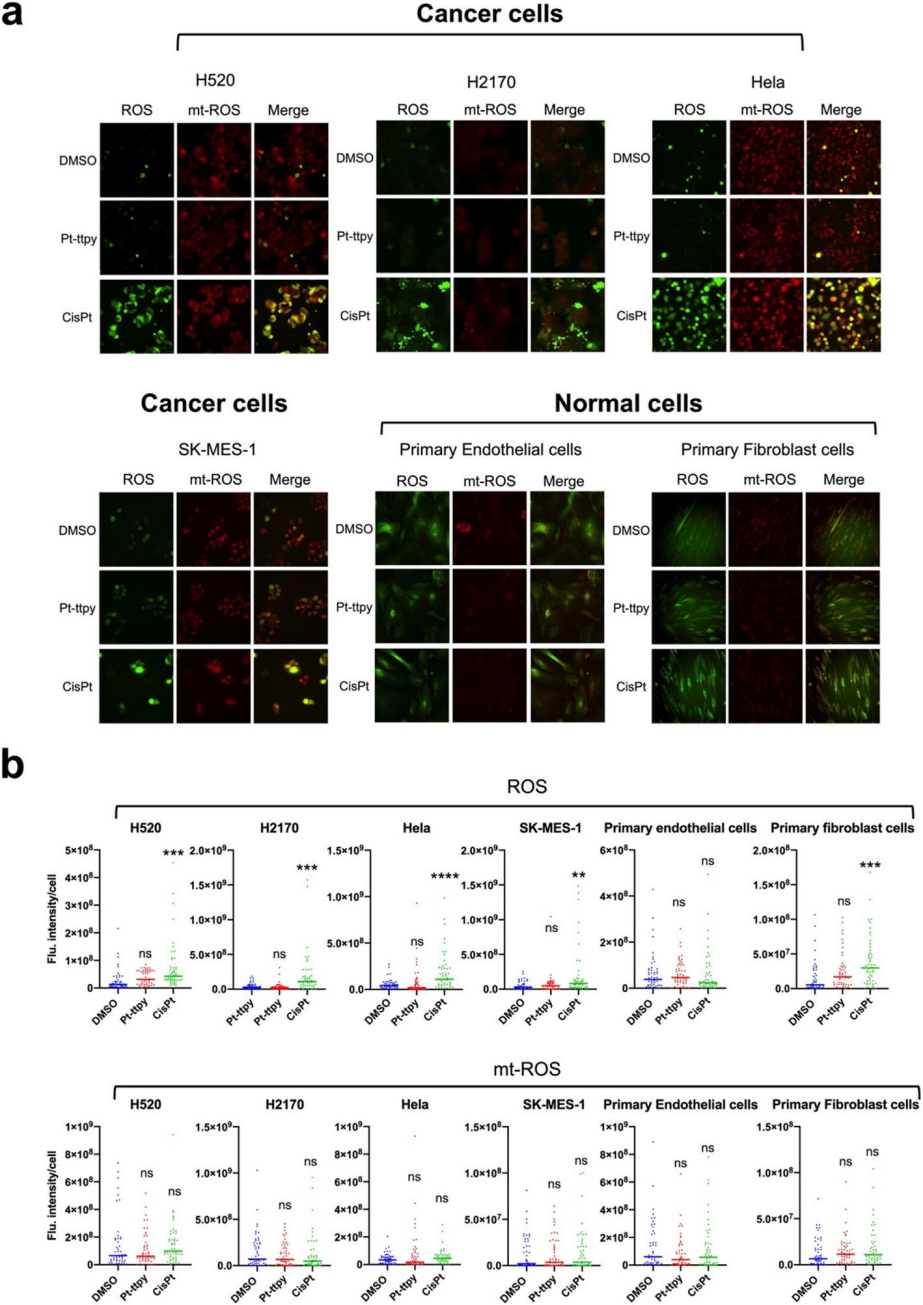
Fluorescence screening of Pt-ttpy and cisplatin effects on ROS and mitochondrial ROS (mt-ROS) induction with single cell fluorescence intensity quantification in four different cancer cell lines and two primary cells (Endothelial cells and Fibroblast cells) post DMSO, Pt-ttpy or cisplatin treatments. **a** represented figures of fluorescent imaging of different cell (line) under Pt-ttpy and cisplatin treatments for 24 h at either 10µM (for cancer cells) or 1µM (for primary cells). The general ROS production was indicated by green colour (ex: 488 nm), the mitochondrial specific ROS (mt-ROS) production was indicated by red colour (ex: 555 nm). **b** The single cell fluorescence quantification for both ROS and mt-ROS in different cell (lines) post indicated treatments was performed by Image J using in-house developed Macros, DMSO group (*n* > 50), Pt-ttpy group (*n* > 50), cisplatin group (*n* > 50). Line indicates the median flu. intensity, P values were calculated toward the DMSO group: *P < 0.05, **P < 0.01, ****P < 0.0001, unpaired t-Student test

Since ROS (Reactive oxygen species) can induce and/or result from mitochondrial dysfunction [42], total cell and mitochondrial ROS (mt ROS) production was quantified by flow cytometry (Fig. 3c and Supplementary Fig. S7 and S8). Consistently with previous works showing that cisplatin’s toxicity on mt relies in part on ROS production that dictates cancer cell fate [43], our present data indicates that cisplatin induces both general and mt ROS production for both 24 h and 96 h treatments (Fig. 3c and Supplementary Fig. S7 and S8). As well, a slight induction of general ROS was observed after Pt-tpy treatment (Fig. 3c). In contrast, Pt-ttpy did not generate any ROS irrespective of the time of treatments and drug concentration (Fig. 3c and Supplementary Fig. S7 and S8). To further study the unique anti-tumor effects of Pt-ttpy distinct from cisplatin in terms of ROS induction, we conducted a screening of Pt-ttpy and cisplatin effects on another four different tumor cell lines (Hela, H520, H2170 and SK-MES-1), two primary cells (endothelial cells and fibroblast cells) and two normal colon epithelial cells (HcoEpic and NCM460). Indeed, the significant induction of ROS in all tumor cells was only observed in the treatment of cisplatin (Fig. 4). Notably for primary cells, we detected also only cisplatin induced a robust production of ROS in the primary lung tissue fibroblast cells and another two normal epithelial cells (Fig. 4 and S9), indicating its potential more side effects to normal tissue, as compared with Pt-ttpy (Fig. 4 and S9). Collectively, in contrast to cisplatin and Pt-tpy, Pt-ttpy disturbs strongly Mitochondrial genome with a significant induction of mt dysfunction indicated by a high reduction of mt membrane potential, oxygen consumption rate and ATP synthesis, and more early apoptotic signals as well as mt morphology switching, but independent of both general and mt-ROS production that is usually involved in platinum-related cell death induction.

**Fig. 5:**
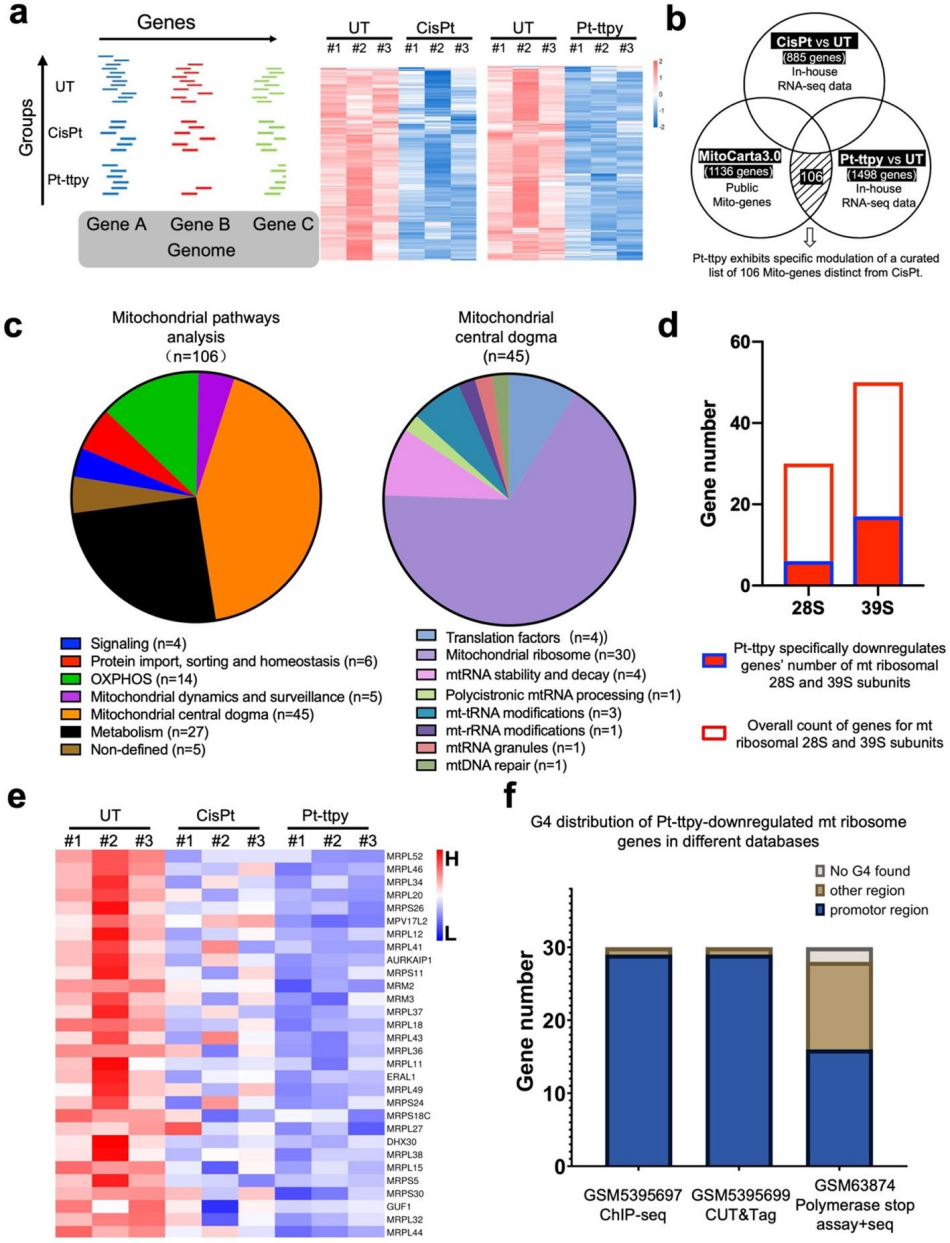
Pt-ttpy show preferable inhibition of mitochondrial ribosome-related gene expression by RNA seq, as compared with cisplatin treatment of A2780 cells for 96h at their IC80 concentration, respectively. **a** Left: Schematic illustration of RNA seq under different treatments, heatmap showcasing the down-regulated gene expression under cisplatin and Pt-ttpy treatments, as compared with the UT group. Each group has three biological replicates. **b** This Venn diagram illustrates 106 mitochondrial genes specifically down-regulated by Pt-ttpy. The detailed process is described in the section of Materials and Methods. **c** Left: Analysis of mitochondrial pathways indicates that Pt-ttpy-specifically down-regulated genes (106 genes) exhibit high enrichment within the mitochondrial central dogma (45/106) [38]. Right: these genes predominantly impact the expression of mitochondrial ribosome genes (30/45). This analysis employed the MitoCarta3.0_MitoPathways tool [38]. **d** Plotting of gene number distribution for mitochondrial ribosome genes specifically down-regulated by Pt-ttpy and the overall count of genes for mitochondrial ribosome 28S and 39S subunits (**e**) A heatmap analysis was conducted to visualize the expression levels of mitochondrial ribosome genes specifically down-regulated by Pt-ttpy in the UT, cisplatin, and Pt-ttpy treatment groups. #1, #2, #3 means the biological replicates for each group. The data was sorted and visualized by raw-normalized values. **f** Pt-ttpy specifically downregulates mt ribosome genes show high enrichment of G4 distribution mostly in the promoter region from various databases [31, 32]

### Pt-ttpy specifically impairs G4 high enriched nuclear-encoded mt ribosome genes’ transcription initiation and elongation

Because we detected that only Pt-ttpy broadly inhibited the protein levels of mt OXPHOS complexes including mt gene-encoded protein CIV-MTCO1 and nuclear gene-encoded protein CIII-UQCRC2/ CIII-Core protein2, CII-SDHB/ CII-30 kDa and CI-NDUFB8 (Fig. 1e and Supplementary Fig. S2), but not CV-ATP5A, we hypothesized that Pt-ttpy may also induce mt dysfunction through indirect effects on nuclear-encoded mt related genes. We therefore performed RNA-seq to study the specific impact of Pt-ttpy effects on nuclear-encoded mt associated genes’ expression as well as on the whole nuclear-encoded (Fig. 5 and S10). To delineate the distinct property of Pt-ttpy in inducing mt dysfunction through mechanisms independent of ROS, we also introduced the setting group of cisplatin for RNA seq (Fig. 5a). At least, the cisplatin-treated group can serve as a valuable reference for understanding cell death induction mechanisms associated but not restricted to ROS production, thereby possibly distinguishing it from the impacts of Pt-ttpy treatment. Consequently, our study was designed to pinpoint gene(s) related to nuclear-encoded mitochondrial proteins that were specifically down-regulated due to Pt-ttpy treatment, and that effects occurs without the induction of ROS. The process of mining the cohort of genes is depicted in the materials and methods section with the sketch shown in Fig. 5b. We successfully identified Pt-ttpy specifically down-regulated 14 nuclear-encoded mt OXPHOS genes from a total of 106 genes with mitochondrial pathways showing specifical down-regulation due to Pt-ttpy treatment (Fig. 5c on the left). Very interestingly, the largest sub-cohort among these 106 genes (comprising 45 genes) is predominantly involved in the mitochondrial central dogma [30], notably impacting the expression of a majority of nuclear-encoded mt ribosome genes (30 genes) (Fig. 5c, d and e). Additionally, the remaining 15 genes are primarily related with mt-RNA modifications and its related translation factors (Fig. 5c on the right). Interestingly, when comparing various methods (polymerase stop assays, BG4-ChIP seq and the latest CUT&Tag seq) employed for different databases detailing G4 distribution with distribution of the mt ribosome genes down-regulated by Pt-ttpy [5, 31, 32], we found that most of mt ribosome genes show high G4 abundance in their promoter region (Fig. 5f). These findings suggest that Pt-ttpy potentially targets mt ribosome genes that are highly enriched in G4 structures within their promoter regions, distinguishing them as unique targets in comparison to cisplatin. In addition, we analyzed the whole transcription pattern following both treatments (Fig. S10). Ontology analysis of GO, KEGG pathway, and potential biological effects indicate that cisplatin predominantly regulates differential genes enriched in ROS metabolism-related pathways (highlighted in yellow, Fig. S10 a/b). Moreover, a significant broad effect of Cisplatin is detected in kidney and heart development (Fig. S10a), which may contribute to Cisplatin-induced side effects, that is already well documented by clinical studies. In contrast, gene ontology analysis does not reveal Pt-ttpy’s influence on ROS-related genes but highlights alterations in cell adhesion-related genes (Fig. S10 d/e).

### Pt-ttpy inhibits the recruiting of TAF1 and NELFB to the nuclear-encoded mt ribosome genes’ promoter and dampens MT ribosome function

**Fig. 6:**
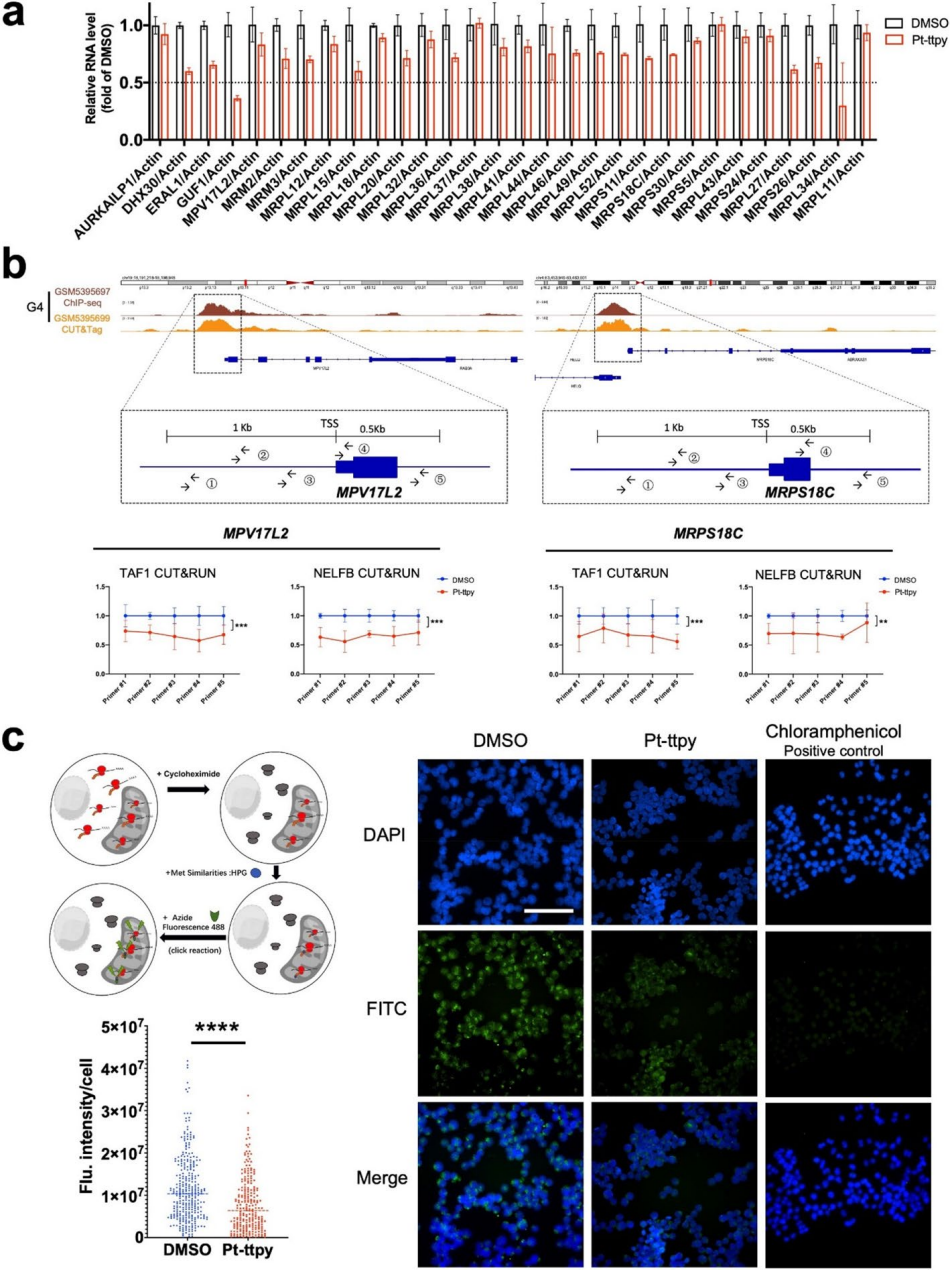
Pt-ttpy impairs G4 high enriched nuclear-encoded mt ribosome genes’ transcription initiation and elongation and dampens specific mt ribosome function of translation in A2780 treated cells. **a** RT-qPCR experiments confirmed Pt-ttpy show a broad inhibition of MT ribosome gene expression. Data is represented as mean ± SEM (*n* = 3). **b** Up: IGV visualization of *MPV17L2* and *MRPS18C* genes containing high abundance of G4 sequences in the TSS regions from the latest DNA G4 databases [32]. And five pairs of primers were designed in around 1.5Kb TSS region and CUT&RUN experiments were performed to detect transcription and elongation factors binding. Down: Pt-ttpy significantly reduced the occupancy of TAF1 (general transcription factor TFIID subunit) and NELFB (Pol II-associated NELF complex member B) at the promoter and its surrounded regions of mt ribosome genes *MPV17L2* and *MRPS18C*. The data were expressed as the mean ± SEM (*n* = 3). P values were calculated by 2way ANOVA analysis between DMSO and Pt-ttpy groups: *P < 0.05, **P < 0.01, ****P < 0.0001. **c** Left up: Schematic illustration of studying flow of mt ribosome function by the Click-chemistry based IF assay. Left: down: Single-cell quantification showed Pt-ttpy significantly inhibited mitochondrial translation, DMSO group (> 200), Pt-ttpy group (*n* > 200). Data represents three independent experiments. Right: represented figures of fluorescent imaging of the mitochondria under Pt-ttpy and DMSO treatments. A Positive control of blocking the synthesis of mitochondrial proteins by chloramphenicol was also presented. Line indicates the median Flu. intensity value, P values were calculated toward the DMSO group: *P < 0.05, **P < 0.01, ****P < 0.0001, unpaired t-Student test

To validate the RNA-seq findings, we conducted RT-qPCR (Fig. 6a) and confirmed that Pt-ttpy exerts a broad inhibiting effects on the expression of mt ribosome genes in A2780 cells.

To decipher the mechanisms of how Pt-ttpy induces the widespread inhibition of nuclear-encoded mt ribosome genes, first, we explored whether Pt-ttpy achieves this by inducing DNA damage within and around the G4-rich regions related with mt ribosome genes. To investigate that, we retrieved our previous work ased on γ-H2AX chromatin immunoprecipitation (ChIP-seq) analysis which suggested that Pt-ttpy induces DNA damage in G-rich regions in A2780 cells on the genomic level (as reported in our prior work [27]. However, upon close examination, we found no evidence of DNA damage within the sequence of any of the mitochondrial ribosome genes’ sequences upon the same treatment with Pt-ttpy. Two represented mt ribosome associated genes’ results are presented in Supplementary Fig. S11.

Next, we questioned if our G4-ligand Pt-ttpy might target the G4-enriched promoter region of mt ribosome genes. This could potentially involve inhibiting the binding of transcription factors (TFs) to their promoters, thus regulating their expression broadly down, because recent works reveal that promoter G4s act as a site for the recruitment of key components of the transcriptional machinery [44], and a reciprocal regulation between native G4 dynamics and gene transcription on genome-wide level by a more sensitive G4-CUT&Tag method [32]. So we established a CUT&RUN-qPCR assay using general transcription factors’ antibodies, and we clearly see Pt-ttpy significantly reduced the occupancy of TAF1 (general transcription factor TFIID subunit) and NELFB (Pol II-associated NELF complex member B) at the specific mt ribosome genes *MRPS18C* and *MPV17L2* promoter and its surrounded regions (Fig. 6b), indicating that Pt-ttpy targets mt ribosome genes’ promoter G4 enriched region and impairs the recruitment of transcription factors to their promoter and its proximal regions.

To further study the consequence of Pt-ttpy’s inhibiting effects of mt ribosome genes expression that would mostly dampen ribosome-mediated translational machine function, we further tested Pt-ttpy effects on mitochondrial specific translation by Click-chemistry-based immunofluorescence (IF) assay with single cell quantification [34], the principle for labeling is presented in Fig. 6c left. Clearly, we detected that Pt-ttpy show a strong inhibition of mitochondrial translation in A2780 cells (Fig. 6c), which is confirmed by another typical cancer cells Hela (Supplementary Fig. S12). Collectively, these data indicate that Pt-ttpy impairs the recruitment of transcription initiation and elongation factors of NELFB and TAF1 in nuclear-encoded mt ribosome genes’ G4 rich promoter region and inhibits their expression broadly with a significant dampening of mt ribosome function.

### Pt-ttpy shows significant anti-tumor effects and presents mitochondrial toxicityin vivowith less side effects, as compared with cisplatin

To further investigate the potential in vivo anti-tumor effects of Pt-ttpy, specifically focusing on its impact on mitochondria, we conducted a study using the A2780 xenograft mouse model to assess the effects of Pt-ttpy and cisplatin.

**Fig. 7:**
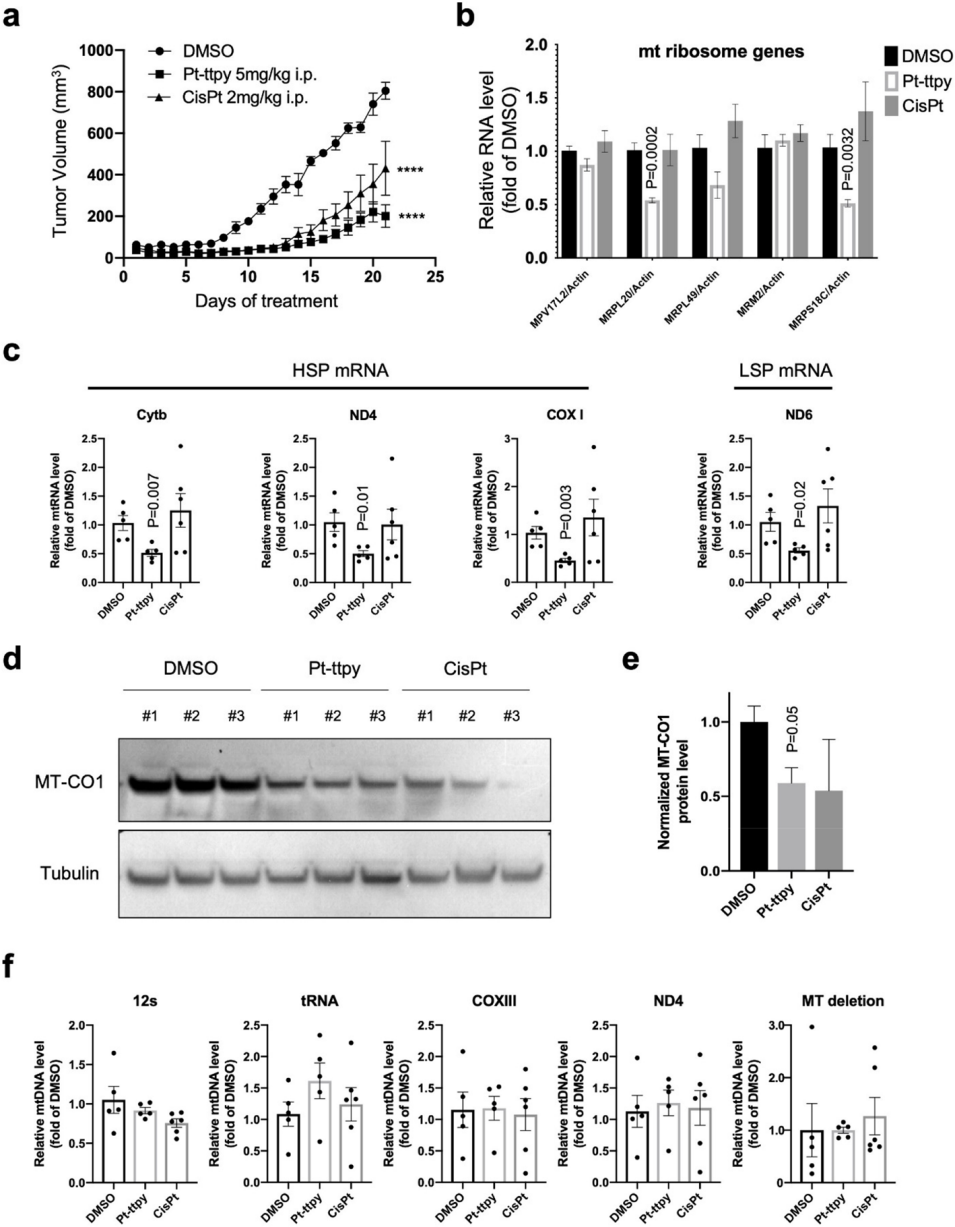
In vivo: A2780 nude mice xenograft tumor model suggests that Pt-ttpy, not cisplatin, significantly inhibits the mRNA levels of both nuclear-encoded mt ribosome genes expression and mt both light and heavy chains-encoded genes and downregulates mt protein MT-CO1 by tumor tissue samples, DMSO group (*n* = 5), Pt-ttpy group (*n* = 5), and cisplatin group (*n* = 6). **a** Tumor growth curves of nude mice under the treatments of DMSO, Pt-ttpy and cisplatin, respectively (**b**) RT-qPCR quantification of mRNA levels of mitochondrial ribosome-related genes in tumor tissues. **c** RT-qQCR quantification of mRNA levels of genes-encoded by both mitochondrial light and heavy chains. **d** Western blot results of MT-CO1 Protein expression levels, *n* = 3. **e** Graph show the quantification of MT-CO1 protein levels after normalizing the data to DMSO group. **f** q-PCR quantification of mt gene copy number changes by different primers located in different mt gene region in tumor tissues. Data are expressed as mean ± SEM of three biological replicates. P values were calculated by unpaired t-test: *P < 0.05, **P < 0.01, ****P < 0.0001

Based on use of cisplatin for in vivo xenografts (intraperitoneal injections at 2 mg/kg) [45], and that Pt-ttpy didn’t show any in vivo toxicities at 5 mg/kg we suggested the intraperitoneal drug administration for Pt-ttpy (5 mg/kg) and cisplatin (2 mg/kg), with treatments administered once every two days over a 21-day period. Our findings revealed that Pt-ttpy exhibited significant anti-tumor effects with reduced toxicity to normal tissues, particularly the kidney and liver, when compared to cisplatin (refer to Fig. 7a and Supplementary Fig. S13 and S14). Cisplatin is renowned for its capacity to induce nephrotoxicity, a condition that significantly compromises kidney function and is closely linked to intracellular stress responses, prominently oxidative stress [46]. We confirmed that cisplatin leads to a decline in liver function, as evidenced by a decrease in the liver index (Supplementary Fig. S13b). Additionally, proximal renal tubular epithelial cells after treatment with cisplatin exhibited turbidity staining and swelling, whereas these effects were less pronounced after treatment with Pt-ttpy (Supplementary Fig. S13c). Concerning the potential liver toxicity caused by all the Pt complexes, cisplatin induced significant inflammation around the portal vein and blood vessels, resulting in enlarged sinusoidal spaces and vascular congestion, effects that were either less prominent or absent in the Pt-ttpy treated group (Supplementary Fig. S14). In terms of potential cardiac toxicity, neither Pt-ttpy nor cisplatin exhibited signs of vascular congestion, fatty degeneration of cardiomyocytes, structural abnormalities, or obvious myocardial rupture phenomena (data not shown). In summary, our studies indicate that Pt-ttpy offers a relatively safer profile compared to cisplatin.

Through RT-qPCR analysis on tumor tissue samples, consistent with our in vitro study, only Pt-ttpy showed significant inhibition of nuclear-encoded mt ribosome genes (Fig. 7b) and mt-encoded genes (Fig. 7c) and protein expression levels (Fig. 7d and e). In contrast with our prior in vitro study, Pt-ttpy did not show clear effects on mt DNA copy number in the i*n vivo* tumor samples (Fig. 7f), which would be explained by the high variation of mt DNA copies in tumor tissues [47]. Collectively, we proposed a model of Pt-ttpy-mediated profound inhibition on mitochondrial genome in cancer cells through a direct effect on mitochondria and an indirect effect based on broad inhibition of G4-enriched nuclear-encoded mt ribosome genes expression.

## Discussion

Given the evidence that mitochondria can be the targets of G4-interactive compounds [8, 9] and platinum complexes [37, 48–50], we envisioned that Pt-ttpy that combines G4-binding properties and a platinum coordinating moiety, may localize in mitochondria and play a significant role in the mechanisms underlying mitochondrial toxicity. In this line, we raised the questions about whether and how this small molecule affects the processes associated with mitochondrial function based on its unique dual properties. To this aim, we performed a comprehensive in vitro and in vivo mechanistic study of Pt-ttpy on both nuclear and mt genome in regulating mt homeostasis and thus explore its potential anti-cancer therapeutic benefits, comparing with two other Pt complexes: a close structural analogue, Pt-tpy that display a weak/non G4-binding property and the prevalent and well-established chemotherapy agent cisplatin. We demonstrated that Pt-ttpy shows a strong disturbance to mitochondrial genome and its function, both in vitro and in vivo. Mechanistic studies suggest Pt-ttpy’s potent dysfunction of mitochondria is related to its direct targeting to mitochondria *via* its high accumulation in mitochondria and indirect targeting to mitochondria through inhibiting mt ribosome associated genes’ expression in chromatin by impairing the recruitment of TF to their G4 rich promoter and proximal regions. Notably, the global impact of Pt-ttpy on mitochondria does not induce ROS production that is, otherwise, typically contributing to platinum complexes treatment-related side effects. Importantly, these data are correlated with in vivo evidence of Pt-ttpy presenting more reduced side effects with effective anti-cancer benefits, as compared with cisplatin.

Increasing evidence supports the ability of mitochondrial DNA (mt DNA) to form G4 in cancer cells, and mt G4 dysregulation affects mt nucleic acid synthesis [12] and mt function as well as mt DNA deletion formation [20]. Interestingly using our G4 ligand Pt-ttpy, we detected its high accumulation in mitochondria with a strong disturbance to mitochondrial genome in cancer cells A2780, whereas its counterpart Pt-tpy with low/no G4 binding property is almost inactive. Pt-ttpy induced mt DNA deletion, interfered with mt replication, transcription, and protein synthesis, leading to mitochondrial dysfunction indicated by mitochondrial membrane potential modification, ATP levels decrease, and markers of mitochondrial damage including a toxic mt morphology switching. Interestingly as compared to another G4 specific ligand RHPS4 used in non-cancerous cells [20], Pt-ttpy does also present direct targeting of mitochondria. Indeed both compounds RHPS4 and Pt-ttpy are lipophilic cations which makes them good candidates for being trapped in mitochondria through strong electrostatic attraction due to the highly negative membrane potential of the inner mitochondrial membrane as is well known [35]. This phenomenon should constitute a first step contributing in part and in a non-specific manner, to the high accumulation of Pt-ttpy (and RHPS4) in mitochondria. Secondly, we suspected that Pt-ttpy, like RHPS4, may directly target mt DNA *via* stabilization of some G4s, since neither Pt-tpy, nor cisplatin, two non-or low- G4 binders, induced a strong effect. Furthermore, we detected a more potent mt DNA lesion for Pt-ttpy that can be attributed to the direct binding of Pt-ttpy to mt DNA through metallic coordination to nucleic bases [23]. Indeed, we can exclude that the mt lesion is due to oxidized guanine [36] since Pt-ttpy does not produce ROS, in contrast to cisplatin [43]. Nevertheless, unlike RHPS4, which appeared to modulate varying levels of mt gene expression, possibly through interactions with the predicted G4 structures in H-stand DNA template in non-cancer cell model [20], Pt-ttpy induces a potent but non-differential inhibition of mitochondrial gene transcription in both the heavy and light strands of mt DNA genes in cancer cells. The disparity may originate, at least, from the fact that epithelial cancer cells exhibit a higher mitochondrial membrane potential (ΔΨm) than their normal counterpart cells [51]. In addition, these differences may arise also from a collecting factors: including the specific cell model employed used with different inherent ability to form G4s [32], the ligands’ capacity to stabilize mt DNA G4 structures, and the distinct treatment protocols applied. A better knowledge of the above factors holds the potential to guide the rational design of personalized anti-cancer treatment strategies of targeting cancer cells’ mt DNA G4.

Importantly, apart from Pt-ttpy direct impact on mitochondria, likely through a G4-dependent mechanism, which triggers a cascade of disruptions in the mitochondrial genome, Pt-ttpy also targets G4 structures in the nuclear genome. It has the potential to influence mitochondrial homeostasis [1]. More specifically, the modulation of G4-rich chromatin regions may conceivably lead to mitochondrial dysfunction. Indeed, nearly around 99% mitochondrial functional proteins are not encoded in mt genome but in nucleus. Notably in this study, we detected that only Pt-ttpy decreased all mt OXPHOS complexes’ protein expression, including CI subunit NDUFB8, CII-30 kDa, CIII-Core protein2 and CIV subunit1, except for CV alpha subunit. Since the proteins down-regulated by Pt-ttpy treatment are not restricted to mitochondrial genome-encoded MTCO1 (CIV subunit1), these data lead us to define alternative G4 forming sequences, beyond mt genome, to decipher the underling mechanism behind Pt-ttpy strong disturbance to mitochondrial function without ROS production, especially as compared with the ROS-related mt toxicity-inducing molecule i.e. cisplatin. Through RNA-seq and Cut&RUN assay using transcription factors’ antibodies, different G4 distribution databases, our work indicates firstly that the promoter region of most mitochondrial ribosomal genes are highly enriched in potential G4 forming sequences, in correlation with the previous finding that G4s in 5’UTR of mRNA coding for ribosomal protein can control their production [52]. Moreover Pt-ttpy, by stabilizing G4, would impair the recruitment of transcription factors to the mt ribosome genes’ G4 forming sequences-related chromatin and decrease mt ribosome genes expression with a functional impairment of mitochondrial ribosome-involved translation process. This work revealed, for the first time on genomic DNA level, that the genes for human mitochondrial ribosomal proteins (MRPs) are targets of the G4 ligand Pt-ttpy. Since increasing data suggested the potential of Mitochondrial Ribosomal Genes as Cancer Biomarkers [53, 54], further investigation is warranted to develop and elucidate the promising anti-cancer effects of the G4 ligand i.e. Pt-ttpy, particularly when targeting selectively overexpressed MRPs in special cancer types.

Considering the abundance of potential binding sites for Pt-ttpy, ranging from 10,000 to 700,000 G4 structures in the human genome, it is highly possible that Pt-ttpy may pose a risk of undesired side effects due to its dual properties targeting on DNA by inducing telomere damage and dysfunction and genomic DNA damage at specific loci. However, we have only demonstrated here that Pt-ttpy induces mitochondrial dysfunction without ROS induction in various cell types, including cancer cells, primary endothelial cells, stromal cells, and normal epithelial cells, consistent with lower toxicity compared to Cisplatin, as observed in our preliminary in vivo studies. Meanwhile, to address potential side effects of Pt-ttpy, particularly its impact on gene transcription levels and subsequent biological effects, we have conducted and assessed Pt-ttpy’s modulation of distinct nuclear genes (refer to Figure S10). Transcriptomic sequencing and gene ontology analysis does not reveal Pt-ttpy’s influence on ROS-related genes in contrast to cisplatin but highlights alterations in cell adhesion-related genes. The question arises whether these differences in nuclear gene expression, particularly those related to cell adhesion (as illustrated in Figure S.10 d/e) due to Pt-ttpy treatments, could lead to potential drug side effects, warranting further toxicological investigation in future.

In terms of mitochondrial dysfunction, cisplatin has been shown to have significant effects on that [43, 55, 56]. One of the mechanisms underlying cisplatin-induced mitochondrial toxicity is the generation of ROS, leading to oxidative stress-induced cell death, which is also a well-defined source of cisplatin-induced side effects [57–59]. In this regard and notably, Pt-ttpy does not function like typical Pt complexes, likely for cisplatin and Pt-tpy, as it neither induces general ROS nor mt specific ROS production in both cancer cell lines and primary tissue cells for short and long time (Figs. 3c and 4 and Supplementary Fig. S7 and S8). This inspiring property of Pt-ttpy-induced mt dysfunction independent of ROS production might be directly correlated with lower toxicity to liver and kidney observed through in vivo studies when compared with cisplatin. Continuing to investigate whether other G4 ligands exhibit similarly to Pt-ttpy-induced mitochondrial dysfunction independent of ROS production, could provide valuable insights into the role of oxidative stress-independent mitochondrial toxicity. This knowledge is also beneficial for the development of platinum-based compounds with enhanced safety profiles.

## Conclusion

This study underscores that, a G4 binding platinum, Pt-ttpy, demonstrates a substantial disruption to the mitochondrial genome function through a direct effect on mitochondria and an indirect effect based on broad inhibition of G4-enriched nuclear-encoded mt ribosome genes expression, spanning from mt DNA replication to its translation in vitro and in vivo. Moreover, we provided the first evidence that most of mt ribosome genes are highly enriched in G4 structures in their promoter regions and thus are the targets of Pt-ttpy that inhibits their expression through dampening the recruitment of TAF1 and NELFB to G4 in nuclear DNA. Lastly, Pt-ttpy displays effective anti-cancer benefits with improved safety, which can be attributed to its induction of significant disruption in mitochondrial function without generation of reactive oxygen species (ROS), thus reducing oxidative stress-related side effects commonly associated with platinum complexes treatments, including cisplatin. Hence, the in vitro and in vivo studies of Pt-ttpy’s activity conducted herein provided us with valuable insights into the therapeutic prospects of drugs targeting mitochondria without generating ROS. Importantly, our work holds strong promise of developing G4-binding platinum-based compounds with improved safety profiles alongside effective anti-cancer benefits.

## Supplementary Information

Supplementary Material 1.

## Data Availability

All data analysed or generated during the study will be included in this published article. Chip-seq data analyzed in this study are publicly available in the Gene Expression Omnibus (GEO) under accession numbers GSE171450. RNA-seq data analysed in this study have already been submitted to GEO and waiting for the confirmation and the accession number. The seq-data is available upon requested, during the reviewing process.
