# Correction: A G-quadruplex-binding platinum complex induces cancer mitochondrial dysfunction through dual-targeting mitochondrial and nuclear G4 enriched genome

**DOI:** 10.1186/s12929-024-01107-5

**Published:** 2024-12-16

**Authors:** Keli Kuang, Chunyan Li, Fatlinda Maksut, Deepanjan Ghosh, Robin Vinck, Maolin Wang, Joel Poupon, Run Xiang, Wen Li, Fei Li, Zhu Wang, Junrong Du, Marie‑Paule Teulade‑Fichou, Gilles Gasser, Sophie Bombard, Tao Jia

**Affiliations:** 1 Key Laboratory of Drug‑Targeting and Drug Delivery System of the Education Ministry and Sichuan Province, Sichuan Engineering Laboratory for Plant‑Sourced Drug and Sichuan Research Center for Drug Precision Industrial Technology, West China School of Pharmacy, Sichuan University, Chengdu, 610041 China; 2 CNRS-UMR9187, INSERM U1196, PSL-Research University, 91405 Orsay, France; 3 CNRS-UMR9187, INSERM U1196, Institut Curie, Universite Paris Saclay, 91405 Orsay, France; 4 Chimie ParisTech, Institute of Chemistry for Life and Health Sciences, Laboratory for Inorganic Chemical Biology, PSL University, CNRS, F‑75005 Paris, France; 5 Hopital Lariboisiere (AP-HP), Laboratoire de Toxicologie Biologique, 2 rue Ambroise Pare, 75475 Paris, France; 6 Department of Thoracic Surgery, Sichuan Clinical Research Center for Cancer, Sichuan Cancer Hospital & Institute, Sichuan Cancer Center, Affiliated Cancer Hospital of University of Electronic Science and Technology of China, Chengdu, China; 7 Department of Medical Oncology, Cancer Center, West China Hospital, Sichuan University, Chengdu, China

**Correction: Journal of Biomedical Science (2024) 31:50** 10.1186/s12929-024-01041-6

Main text

After publication of the article [1], it was brought to our attention that the figure captions of Figure 1 to Figure 5 were mismatch. The correct figure captions of the figures are shown below:

Figure 1. Impact of different platinum (Pt) complexes (cisplatin, Pt-ttpy and Pt-tpy) on cellular uptake and distribution with the potential toxicity to mitochondrial genome at their IC80 concentration in A2780 treated cells. **a** Schematic illustration of platinum quantification flow in cell pellets, genomic DNA and mitochondria is presented in the left, comparative quantification of Pt amount(ng)/5 × 106 cells for cisplatin, Pt-ttpy and Pt-tpy was performed in cell pellet, extracted genomic DNA and isolated mitochondria, respectively after 96 h treatment. Data represents three independent experiments with the mean ± SEM. **b** A sketch of describing different primers’ position in non-deleted mt-DNA, deleted mt-DNA and total mt-DNA is presented, that is used for qPCR analysis as presented in figure **c**. **c** qPCR quantification of different mt-DNA copy numbers under different Pt complexes treatments after 96 h treatment, data is presented as relative fold changes of mtDNA copy numbers for different Pt complexes’ treatment groups compared to the untreated (UT) group. Data represents three independent experiments with the mean ± SEM. **d** RT-qPCR quantification of different mt-RNA levels, including both mt non-protein coding genes and its protein coding genes in response to different Pt complexes’ 96 h treatment groups compared to the UT group. Data represents three independent experiments with the mean ± SEM. **e** Western blot study of different mt OXPHOS complex proteins in the 96 h treatment of different Pt complexes. Also shown is a blot of actin as a loading control. The corresponding quantification data of different mt OXPHOS complex protein levels is presented in the supplementary Fig. 2. Data represents two independent experiments.

Figure 2. Impact of three platinum (Pt) complexes (cisplatin, Pt-ttpy and Pt-tpy) in mitochondrial number and its morphology of A2780 treated cells. **a** Flow cytometry analysis of mitochondrial number changes in the treatment of different Pt complexes by the staining of TOMM20, plotted are the TOMM20 signal distribution in different treatments. The histogram is represented by two independent experiments. **b** Confocal microscope tested the mitochondrial abundance and its morphology changes following the Pt complexes treatments (cisplatin, Pt-ttpy and Pt-tpy) for 96 h. Scale bar: 10 µm. Data represents three independent experiments.

Figure 3. Impact of three platinum (Pt) complexes (cisplatin, Pt-ttpy and Pt-tpy) on mitochondrial homeostasis in A2780 treated cells. **a** Left is presented as the seahorse XF cell mito stress test profile under different Pt complexes treatments (10 µM, 24 h treatment) as well as UT group with specific electron transport chain inhibitors: oligomycin (inhibitor of ATP synthase (complex V)), FCCP (uncoupling agent), antimycin-A (complex III inhibitor), and rotenone (complex I inhibitor). Right is plotted as the quantification of basal respiration, ATP production and spare respiratory capacity respectively by different treatments of Pt complexes. **b** Flow cytometry was used to quantify mitochondrial potential changes by the staining of JC1, % Cells with mitochondrial membrane loss (dysfunctional mitochondria) corresponding to the % of cells with JC-1 in its green monomers form after treatment at the respective IC50 and IC80 concentrations of the complexes. Data represents three independent experiments with the mean ± SEM. **c** Flow cytometry was used to quantify the total ROS production in A2780 cells treated, normalized ROS production is plotted as the mean ± SEM, data represents three independent experiments. P values were calculated toward the UT: *P < 0.05, **P < 0.01, ****P < 0.0001, unpaired t-Student test.

Figure 4. Fluorescence screening of Pt-ttpy and cisplatin effects on ROS and mitochondrial ROS (mt-ROS) induction with single cell fluorescence intensity quantification in four different cancer cell lines and two primary cells (Endothelial cells and Fibroblast cells) post DMSO, Pt-ttpy or cisplatin treatments. **a** Represented figures of fluorescent imaging of different cell (line) under Pt-ttpy and cisplatin treatments for 24 h at either 10 µM (for cancer cells) or 1 µM (for primary cells). The general ROS production was indicated by green colour (ex: 488 nm), the mitochondrial specific ROS (mt-ROS) production was indicated by red colour (ex: 555 nm). **b** The single cell fluorescence quantification for both ROS and mt-ROS in different cell (lines) post indicated treatments was performed by Image J using in-house developed Macros, DMSO group (n > 50), Pt-ttpy group (n > 50), cisplatin group (n > 50). Line indicates the median flu. intensity, P values were calculated toward the DMSO group: *P < 0.05, **P < 0.01, ****P < 0.0001, unpaired t-Student test.

Figure 5. Pt-ttpy show preferable inhibition of mitochondrial ribosome-related gene expression by RNA seq, as compared with cisplatin treatment of A2780 cells for 96h at their IC80 concentration, respectively. **a** Left: Schematic illustration of RNA seq under different treatments, heatmap showcasing the down-regulated gene expression under cisplatin and Pt-ttpy treatments, as compared with the UT group. Each group has three biological replicates. **b** This Venn diagram illustrates 106 mitochondrial genes specifically down-regulated by Pt-ttpy. The detailed process is described in the section of Materials and Methods. **c** Left: Analysis of mitochondrial pathways indicates that Pt-ttpy-specifically down-regulated genes (106 genes) exhibit high enrichment within the mitochondrial central dogma [(45/106) (38]. Right: these genes predominantly impact the expression of mitochondrial ribosome genes (30/45). This analysis employed the MitoCarta3.0_MitoPathways tool [38]. **d** Plotting of gene number distribution for mitochondrial ribosome genes specifically down-regulated by Pt-ttpy and the overall count of genes for mitochondrial ribosome 28S and 39S subunits **e** A heatmap analysis was conducted to visualize the expression levels of mitochondrial ribosome genes specifically down-regulated by Pt-ttpy in the UT, cisplatin, and Pt-ttpy treatment groups. #1, #2, #3 means the biological replicates for each group. The data was sorted and visualized by raw-normalized values. **f** Pt-ttpy specifically downregulates mt ribosome genes show high enrichment of G4 distribution mostly in the promoter region from various databases [31, 32].

The original publication has been updated.
