# Supplementary material for: A G-quadruplex-binding platinum complex induces cancer mitochondrial dysfunction through dual-targeting mitochondrial and nuclear G4 enriched genome
Source: J Biomed Sci. 2024 May 13;31:50. doi: 10.1186/s12929-024-01041-6 (PMC11089687; doi:10.1186/s12929-024-01041-6)
Supplement: Supplementary file 1 — Supplementary Material 1. [file 12929_2024_1041_MOESM1_ESM.docx]

**Supporting Information for**

**A G-quadruplex-binding platinum complex induces cancer mitochondrial dysfunction through dual-targeting mitochondrial and nuclear G4 enriched genome**

**AUTHORS**

Keli Kuang^1†^, Chunyan Li^1†^, Fatlinda Maksut^2, 3^, Deepanjan Ghosh^2, 3^, Robin Vinck^4^, Maolin Wang^1^, Joël Poupon^5^, Run Xiang^6^, Wen Li^7^, Fei Li^1^, Zhu Wang^1^, Junrong Du^1^, Marie-Paule Teulade-Fichou^2, 3^, Gilles Gasser^4^, Sophie Bombard^2, 3^* and Tao Jia^1, 2, 3^*

1. Key Laboratory of Drug-Targeting and Drug Delivery System of the Education Ministry and Sichuan Province, Sichuan Engineering Laboratory for Plant-Sourced Drug and Sichuan Research Center for Drug Precision Industrial Technology, West China School of Pharmacy, Sichuan University, Chengdu 610041, China.
2. CNRS-UMR9187, INSERM U1196, PSL-Research University, 91405 Orsay, France
3. CNRS-UMR9187, INSERM U1196, Université Paris Saclay, 91405 Orsay, France
4. Chimie ParisTech, PSL University, CNRS, Institute of Chemistry for Life and Health Sciences, Laboratory for Inorganic Chemical Biology, F-75005 Paris, France.
5. Hôpital Lariboisière (AP-HP), Laboratoire de Toxicologie Biologique, 2 rue Ambroise Paré, 75475 Paris, France
6. Department of Thoracic Surgery, Sichuan Clinical Research Center for Cancer, Sichuan Cancer Hospital & Institute, Sichuan Cancer Center, Affiliated Cancer Hospital of University of Electronic Science and Technology of China, Chengdu, China.
7. Department of Medical Oncology, Cancer Center, West China Hospital, Sichuan University, Chengdu, China

* To whom correspondence should be addressed: Tao Jia* , Tel: +86 191 5012 0635; Fax: 610041; Email: [taojia86@scu.edu.cn](mailto:taojia86@scu.edu.cn). Correspondence could also be addressed to Sophie bombard* , Tel: +33 1 69 86 31 89; Fax: 91401; Email: [Sophie.bombard@curie.fr](mailto:Sophie.bombard@curie.fr).

† Joint Authors: Keli Kuang^†^ and Chunyan Li^†^

**This PDF file includes:**

Supporting text

Figures S1 to S14

Tables S1

SI References

Supporting Information Text

Materials and Methods

**qPCR-based method for detection of the mt DNA lesion**

Investigating mitochondrial DNA (mtDNA) damage in the treatment of different metallic complexes at their IC_80_ concentrations for 96 hours was performed as previously described protocol (1). Generally, total DNA was purified using DNA Blood and Tissue Kit (Qiagen, Germany) from cells under indicated drug treatments. DNA quantity was determined by NanoDrop (Themo Fisher). The isolated DNA showed a high purity (A260/ A280>1.8) and was stored at -20°C. The primers used for real time amplification were synthesized and HPLC-purified by Eurogentec. The primers used in this study are the ones allowing to detect mtDNA lesion in the region opposite to the D-loop which is a partially relaxed structures and consequently more fragile than other mt-DNA region: Short amplicon primers Forward: CATGCCCATCGTCCTAGAAT, Short amplicon primers Reverse: ACGGGCCCTATTTCAAAGAT; Long amplicon primers Forward: CATGCCCATCGTCCTAGAAT, Long amplicon primers Reverse: TGTTGTCGTGCAGGTAGAGG. Briefly, the PCR conditions to run long and short fragments by QuantStudio 5 real-time PCR system and the mt-DNA damage calculated as lesion per 10 kb DNA of each mt-DNA region were performed in the same manner as previously reported (1). The real-time-PCR amplification of mitochondrial DNA fragments used to detect mtDNA lesions is sensitive enough to detect 8 oxo-G induced by ROS. Consequently, the mtDNA lesions detected by cisplatin treatment could be the consequence of ROS production. In contrast, for the Pt-ttpy treatment, in the absence of ROS production, we can suggest that mtDNA lesions could be attributed to direct Pt-ttpy adducts.

**Mitochondrial membrane potential assay (JC-1 assay)**

Changes in the mitochondrial potential were detected by 5,50,6,60-tetrachloro-1,10,3,30-tetra ethylbenzimidazolylcarbocyanine iodide/chloride (JC-1, Biotium), a cationic dye that exhibits potential dependent accumulation in mitochondria, indicated by fluorescence emission shift from red (590 nm) to green (525 nm). A2780 cells were treated with various concentrations of cisplatin, Pt-ttpy or Pt-tpy. After 96 hours treatment, cells were re-suspended 0.5 mL of PBS containing 2 µM final concentration of JC-1 probe and incubated at 37°C for 15 min. After washing, cells were re-suspended in PBS and analysis was performed on a BD FACSCanto II cytometer. Mitochondria containing red JC-1 aggregates in healthy cells are detectable in the PE or PI channel, and green JC-1 monomers in apoptotic cells are detectable in FITC channel.

**Annexin V apoptosis detection assay with 7-AAD**

The A2780 cells were plated in a 6-well plate (100 000 cells) with indicated metallic complexes Pt-ttpy cisplatin and Pt-tpy at their IC_80_ concentration for 96 hours. A2780 cell death has been assessed by Annexin V-FITC and 7-AAD incorporation (Biolegend) according to the manufacturer’s instructions. Flow cytometry acquisitions were obtained on a FACSCanto IITM analyzer (BD Biosciences) with the assistance of BD FACSDiva Software (BD Biosciences) and data analyzed with the FlowJo Software (Tree Star).

**Statistical analysis**

The data were analyzed using Graphpad Prism 9.0 software (San Diego, CA). The results were presented as either mean ± SEM or ± SD as indicated, details of regarding the number of experimental replicates and statistical analyses methods were indicated in the figure legends.

Figures and Table

**
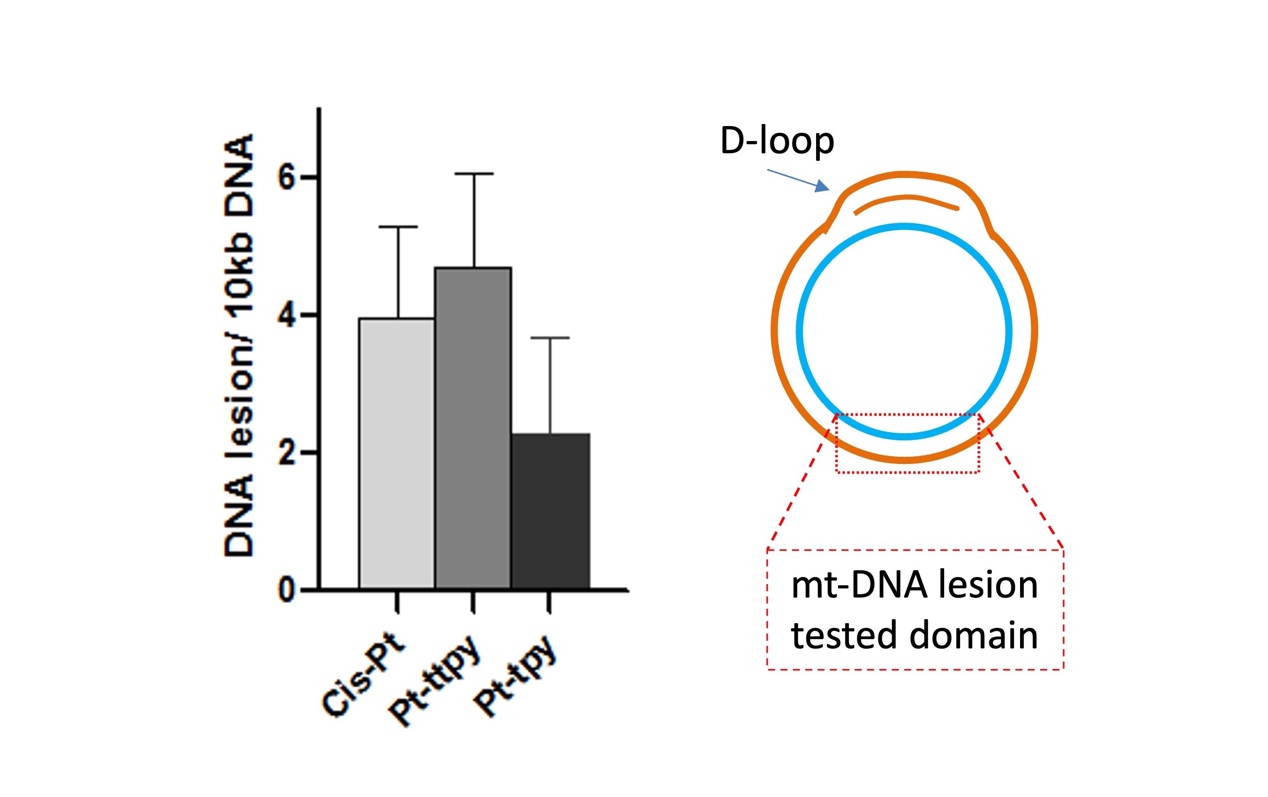
**

**Fig. S1.** Pt-ttpy does not induce significant more DNA damage in mitochondrial genome by a semi-long run real-time PCR approach (1). Quantification of mitochondrial DNA lesion per 10 kb DNA by SLR rt-PCR amplification of total DNA isolated from A2780 cells treated by Pt-ttpy, Pt-tpy and cisplatin at their IC80 con. for 96h in the indicated domain.


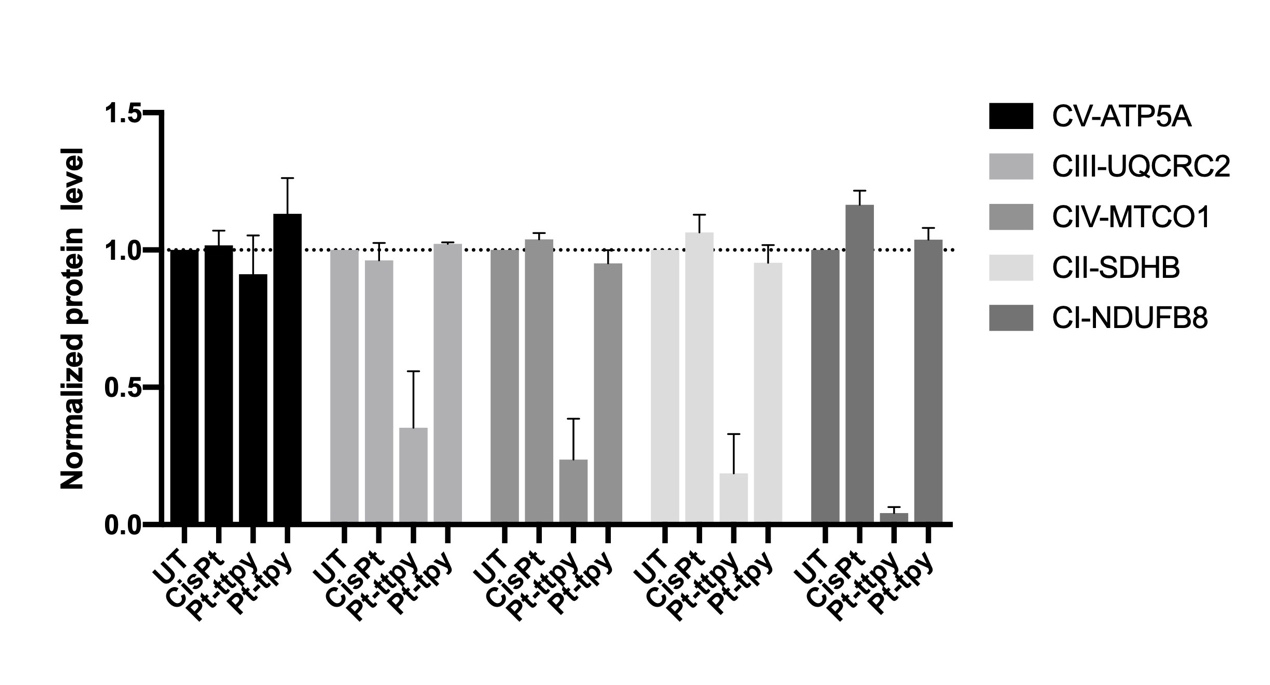
**Fig. S2.** Protein quantification of different MT OXPHOS complex proteins in the treatment of A2780 with the three Pt complexes (cisplatin, Pt-ttpy and Pt-tpy). Data is represented by two independent experiments with mean ± SEM.


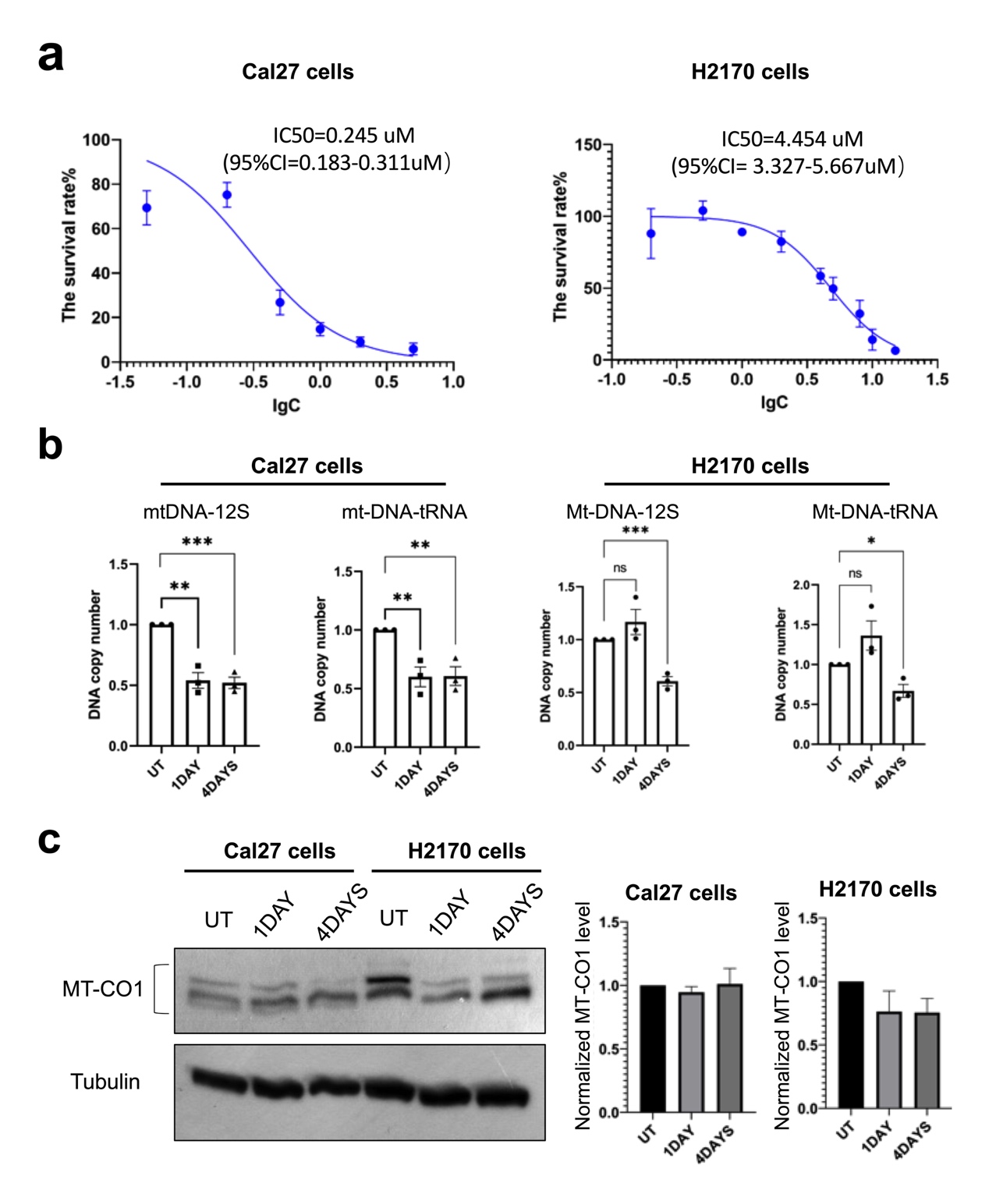


**Fig. S3.** Impact of Pt-ttpy effects on mitochondrial DNA copy number and mitochondria-encoded protein MT-CO1 protein level. (a) IC50 determination of Pt-ttpy in cancer cells Cal27 and H2170 respectively. Data represents three independent experiments with the mean ± SEM (b) qPCR quantification of different mt-DNA copy number changes under Pt-ttpy treatment in Cal27 and H2170 cells at their respective IC80 concentration after 1day and 4days treatments, data is presented as relative fold changes of mtDNA copy numbers for Pt-ttpy group compared to the untreated (UT) group. Data represents three independent experiments with the mean ± SEM. P values were calculated toward the UT: *P < 0.05, **P < 0.01, ****P < 0.0001, unpaired t-Student test. (c) left: western blot study of MT-CO1 protein in the 24h and 96 h treatment of Pt-ttpy at their respective IC80 concentration. Also shown is a blot of tubulin as a loading control. Right: protein quantification of MT-CO1 protein by Image J. Data is represented by two independent experiments. Data represents two independent experiments with mean ± SEM.


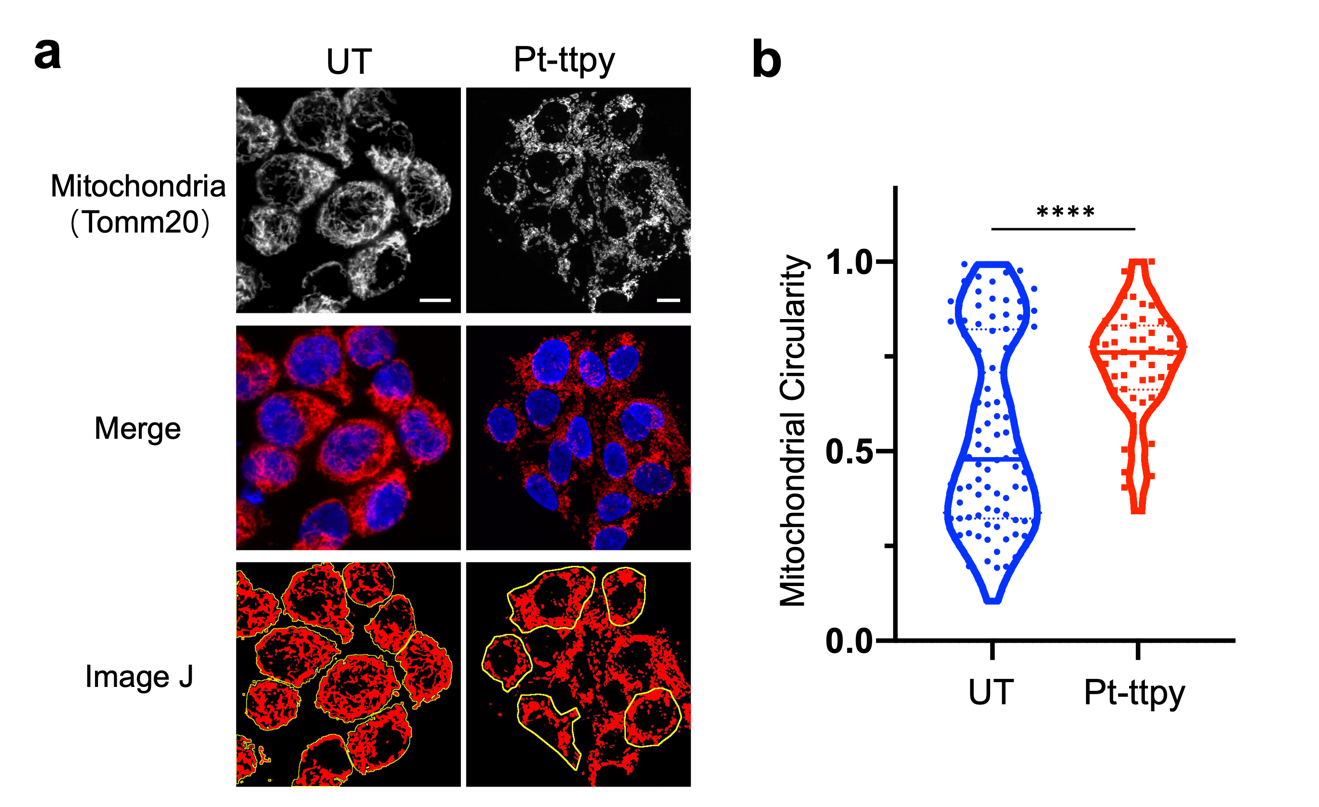


**Fig. S4.** Confocal microscopy detection and quantitative analysis of mitochondrial morphology changes in A2780 cells after treatment with Pt-ttpy at its IC80 con. for 96 hours. (a) Representative confocal and Image J-processed images. Scale bar: 10 μm. (b) Quantitative analysis of mitochondrial morphology changes indicated by circularity value, where the value closer to 1 indicates a perfect circle and the value closer to 0 indicates an elongated shape, at least 50 foci/group was quantified. Data is represented by two independent experiments. P values were calculated toward the UT, ****P < 0.0001, unpaired t-Student test. The horizontal line represents the median, while the dotted lines represent the first quartile and third quartile, respectively.


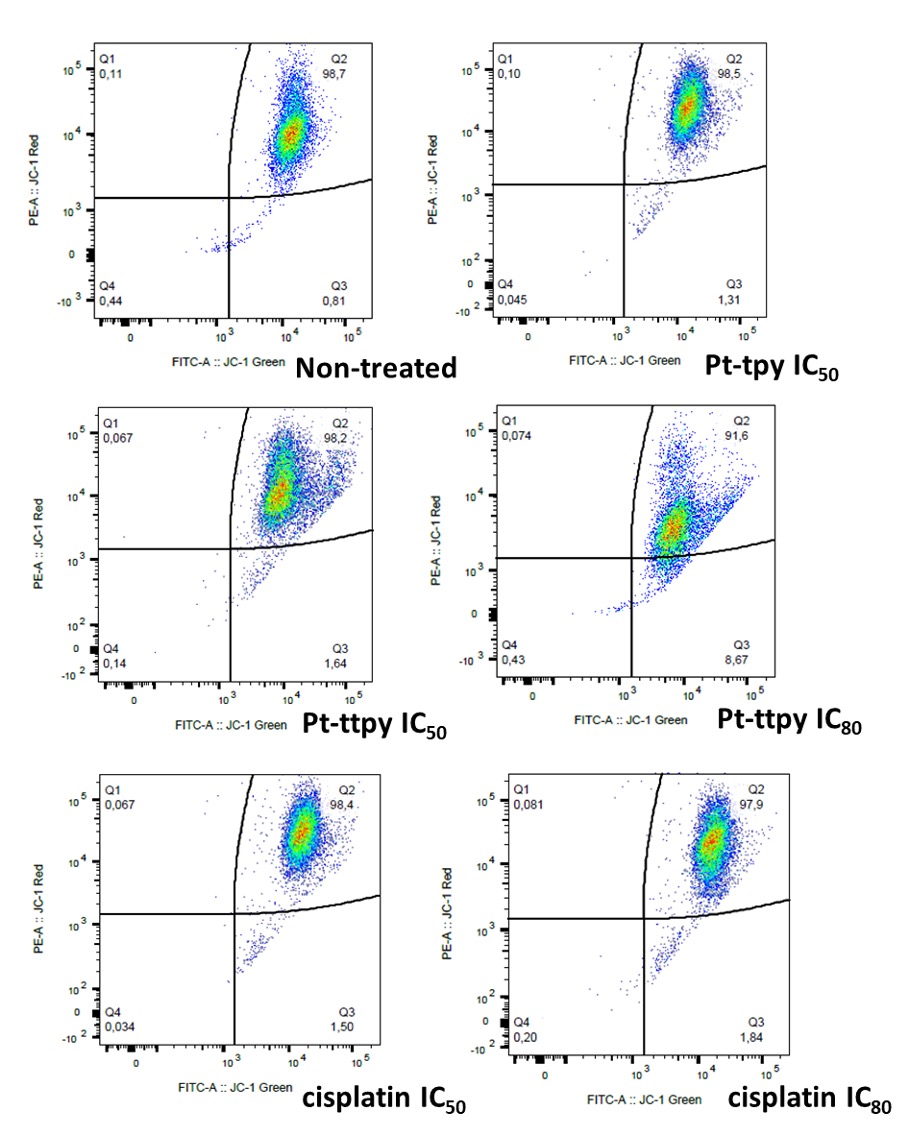


**Fig. S5.** Flow cytometry was used to quantify mitochondrial potential changes by the staining of JC1. Cell population in different quadrants with Q3 representing mitochondrial membrane potential loss (green monomer JC-1) in Pt-tpy, Pt-ttpy and cisplatin treated cells at their IC50 and IC80 concentration for 96 hours in A2780 cells.


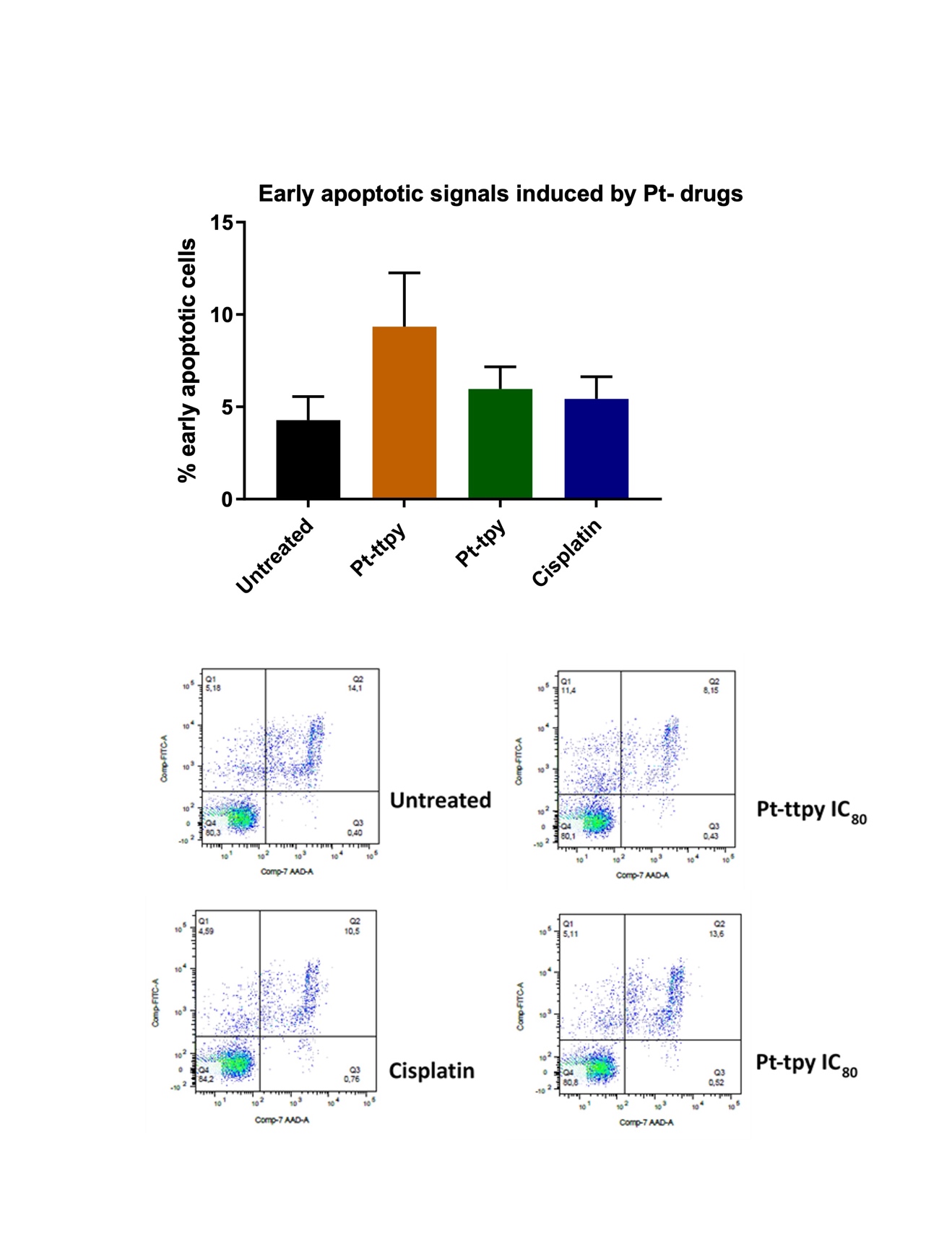


**Fig. S6.** Flow cytometry of the Annexin V apoptosis assay showing an increase of cells in Q1 (FITC+/7-AAD-) for early apoptosis after treatments with the three Pt complexes at their IC80 concentration for 96h. Data are expressed as mean ± SD of two biological replicates.


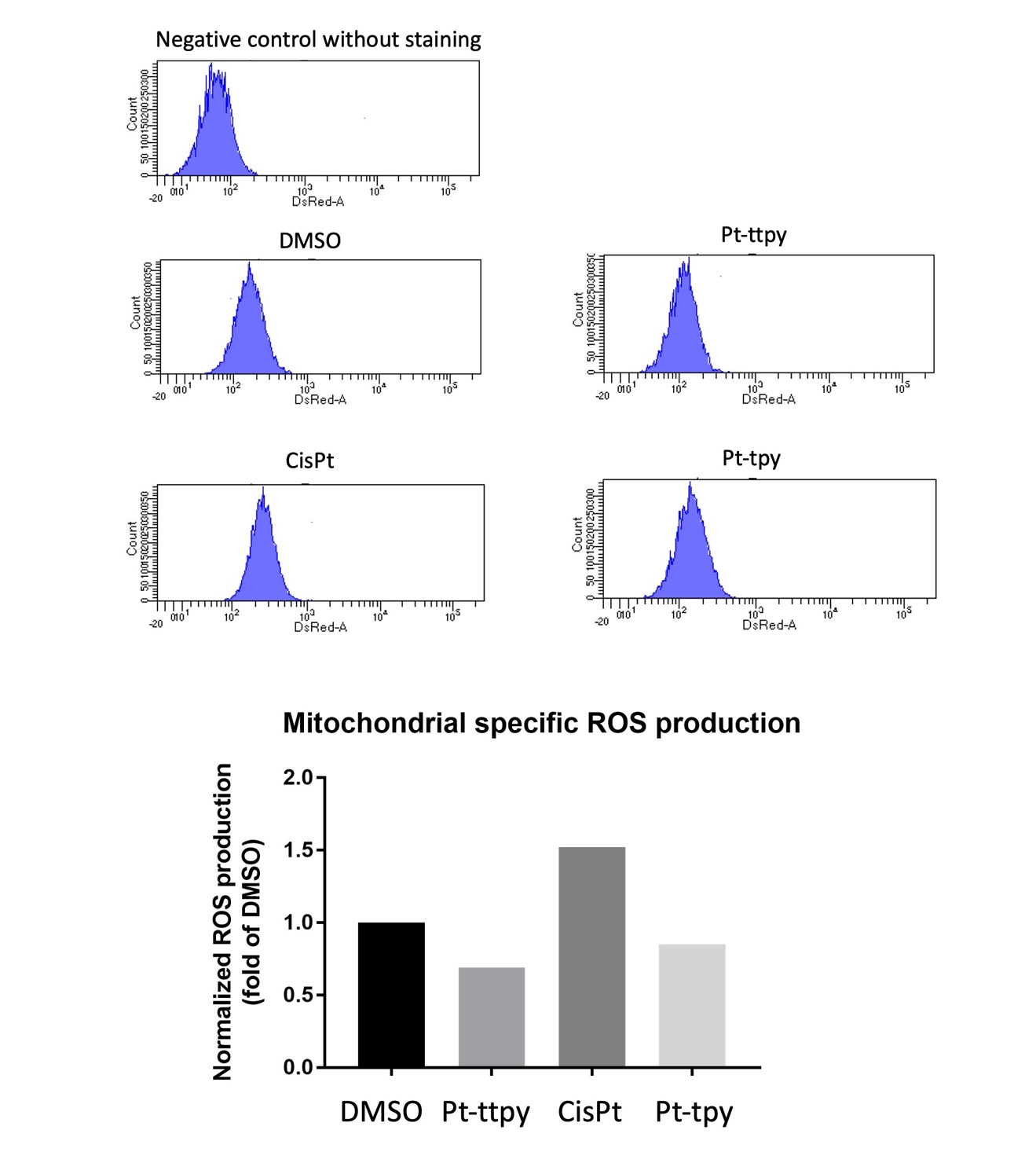
**Fig. S7.** Flow cytometry analysis of mitochondrial specific ROS production under the treatments of A2780 with the three Pt complexes. complexes (cisplatin, Pt-ttpy and Pt-tpy) at their IC80 con. for 96h.


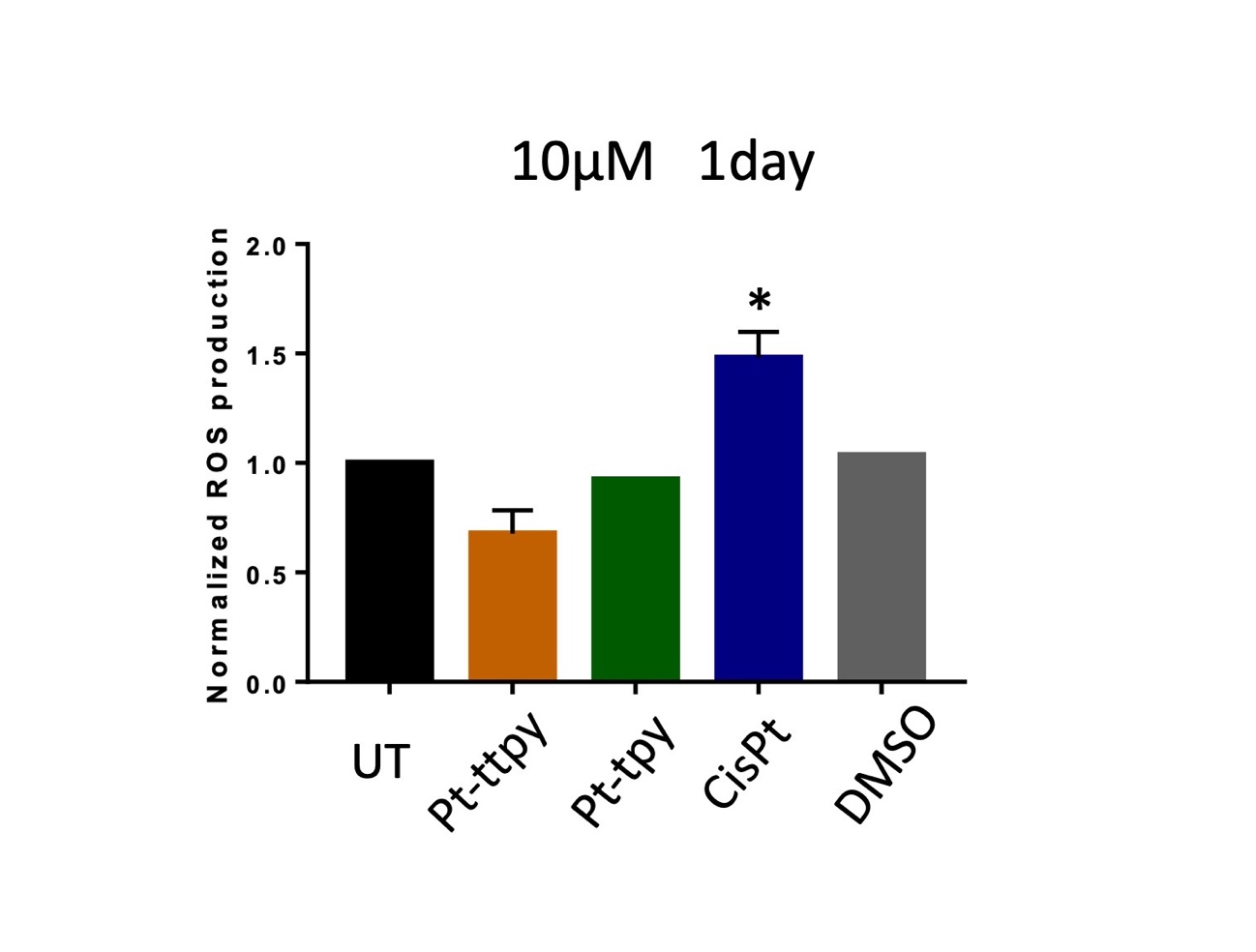


**Fig. S8** Flow cytometry analysis of general ROS production under the treatments of of A2780 with the three Pt complexes (cisplatin, Pt-ttpy and Pt-tpy) at 10µM con. for 24h. Data are expressed as mean ± SEM of three biological replicates. P values were calculated by unpaired t-test: *P < 0.05.


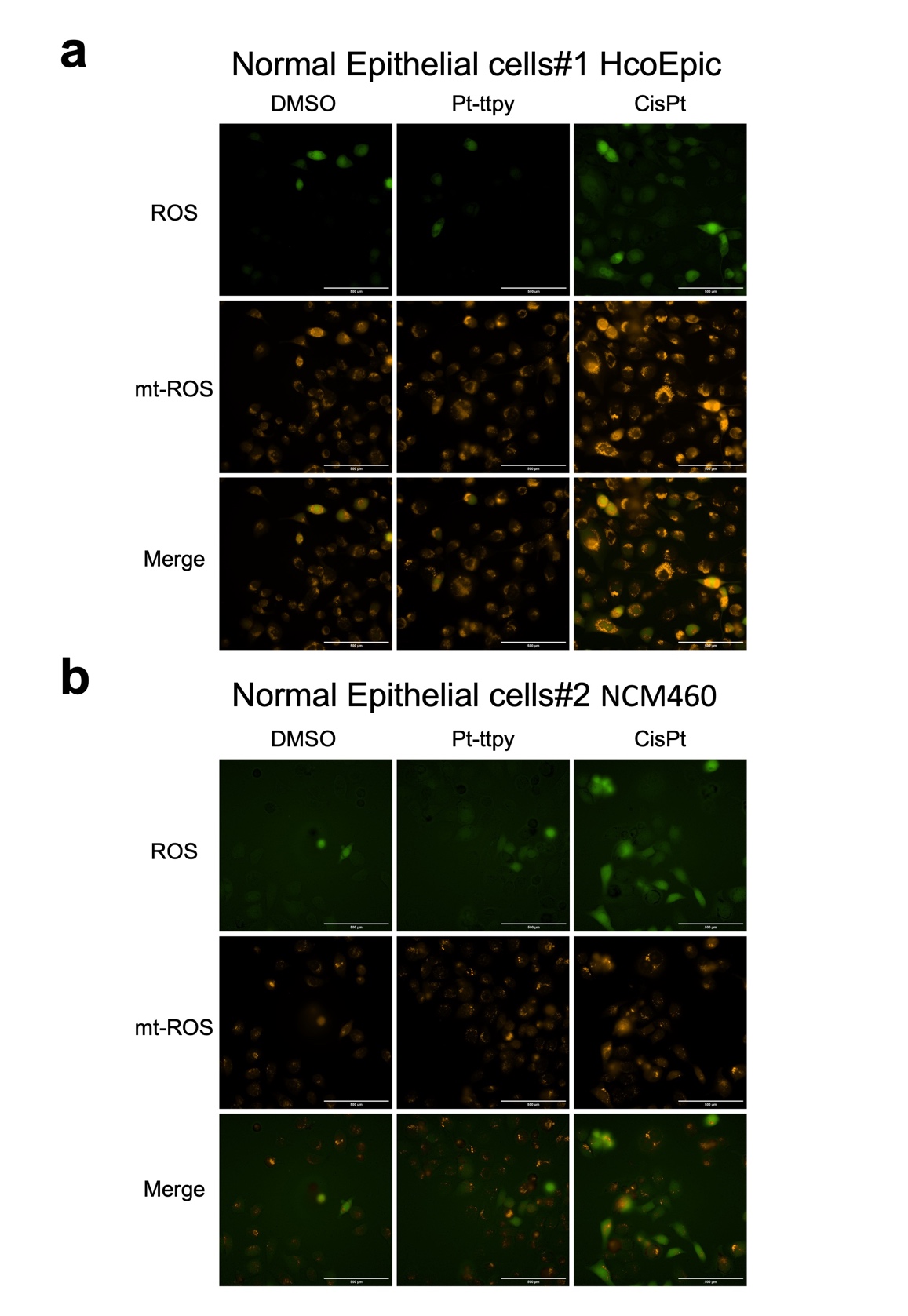


**Fig. S9** Immunofluorescence detection of the effects of 1 μM Pt-ttpy and 1 μM CisPt for 24 hours on ROS and mt-ROS production in normal epithelial cells. (a) Representative images of HcoEpic human normal colon epithelial cells. (b) Representative images of NCM460 human normal colon epithelial cells. The general ROS production was indicated by green colour (ex: 488nm), the mitochondrial specific ROS (mt ROS) production was detected by yellow colour (ex: 555nm). Scale bar: 500 μm.


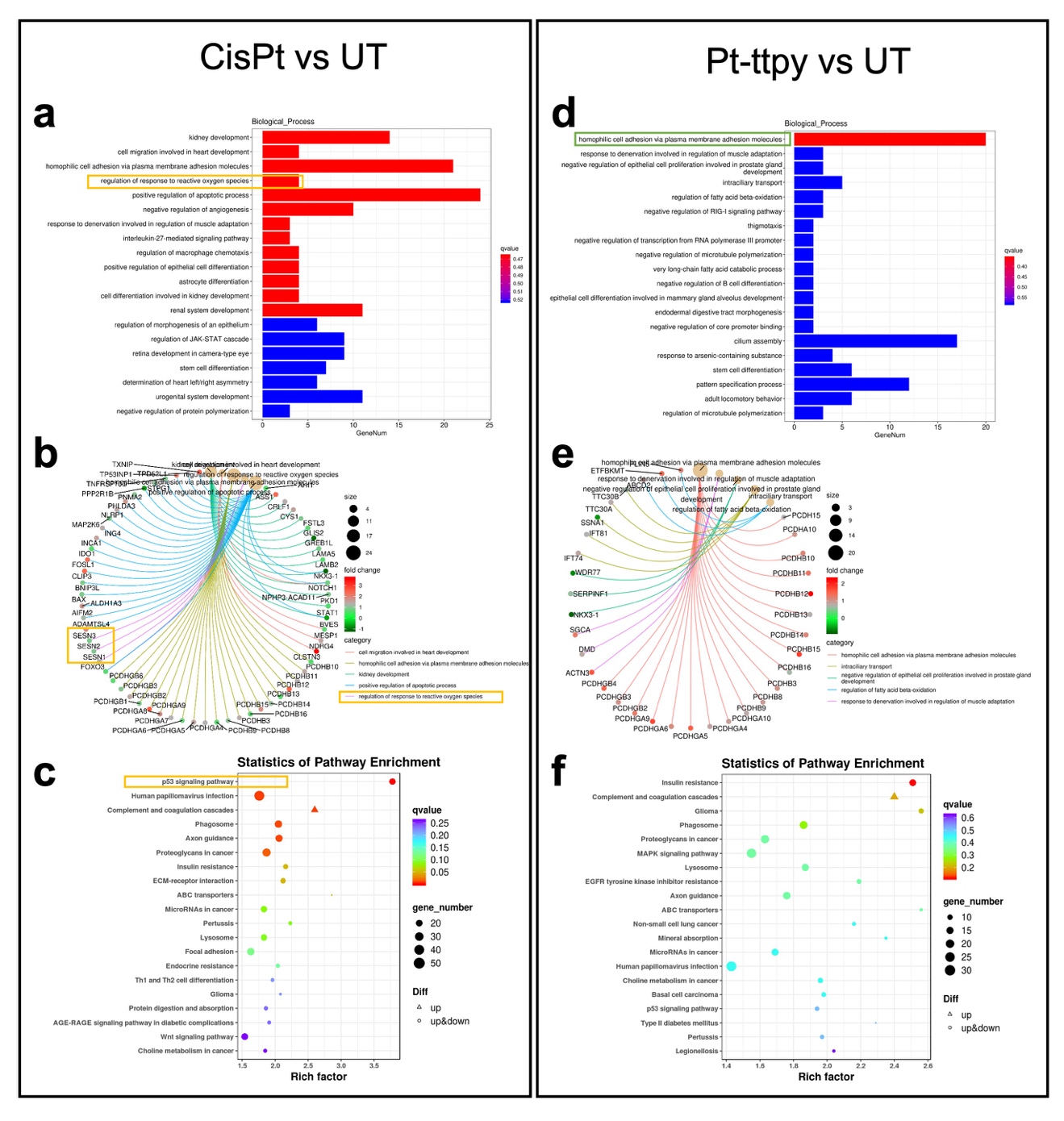


**Fig. S10** Functional enrichment analysis of differentially expressed genes (DEGs) in the CisPt group and Pt-ttpy group by transcriptomics analysis on nuclear genes (FDR<0.05, FC>1.5). (a) Gene Ontology (GO) analysis of DEGs in the CisPt group reveals that, in addition to enrichment in cell adhesion, they are also closely associated with ROS regulation-related biological processes (BP, highlighted in yellow). (b) The network plots of BP enrichment analysis display ROS regulation-related genes in the CisPt group (highlighted in yellow). (c) KEGG pathway enrichment analysis of the CisPt group indicates the most enriched DEGs in the p53 signaling pathway (highlighted in yellow), which is supposed to be involved in DNA damage protection. (d) GO analysis of DEGs in the Pt-ttpy group indicates the highest enrichment of biological processes related to cell adhesion (highlighted in Green). (e) The network plots of BP enrichment analysis in the Pt-ttpy group. (f) The KEGG pathway enrichment analysis of the Pt-ttpy group.


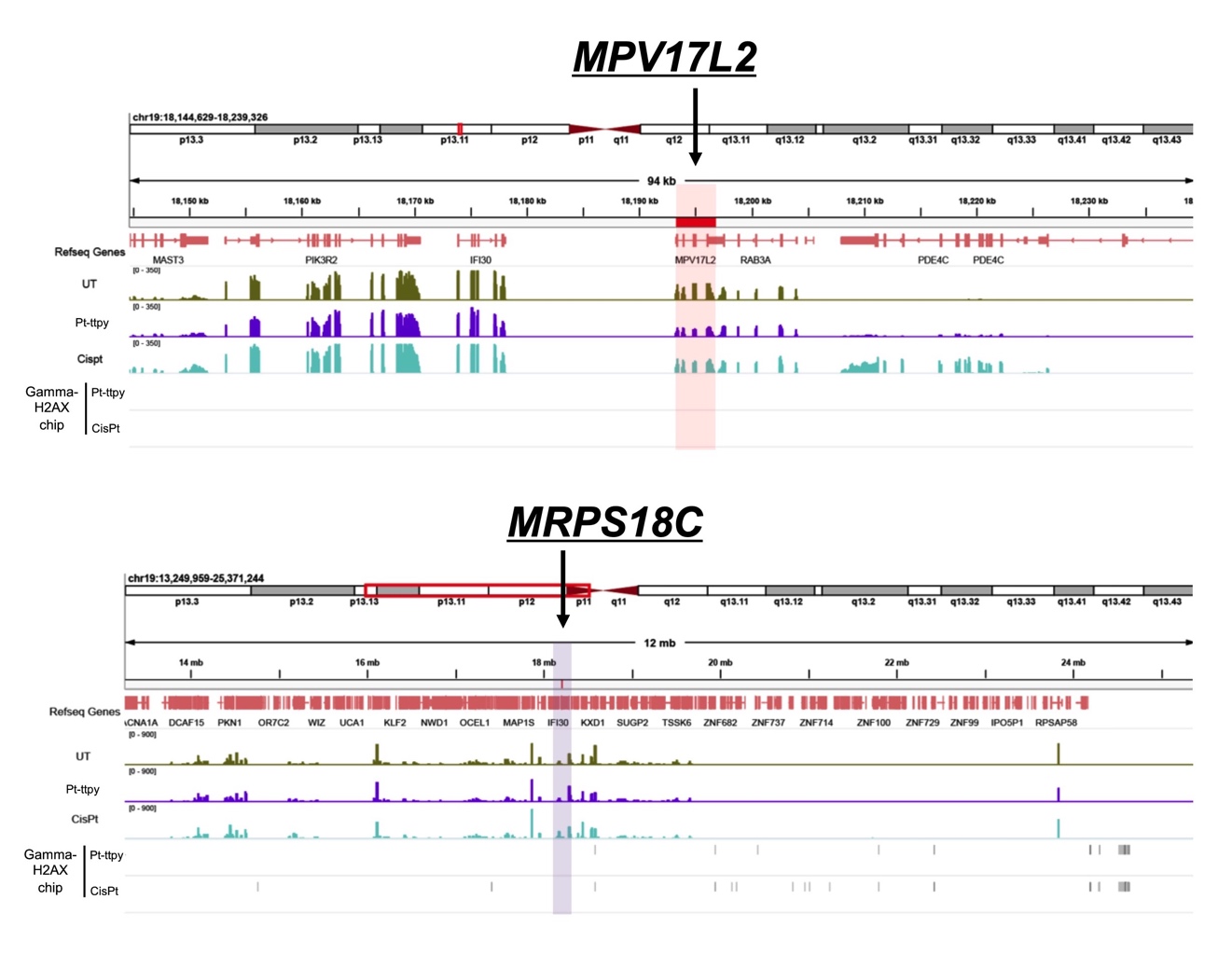
**Fig. S11.** IGV showing the mt ribosome-related genes MRPS18C and MPV17L2 expression, as well as the peak distribution of Pt-ttpy and cisplatin γ-H2AX IPs over the untreated γ-H2AX IP.


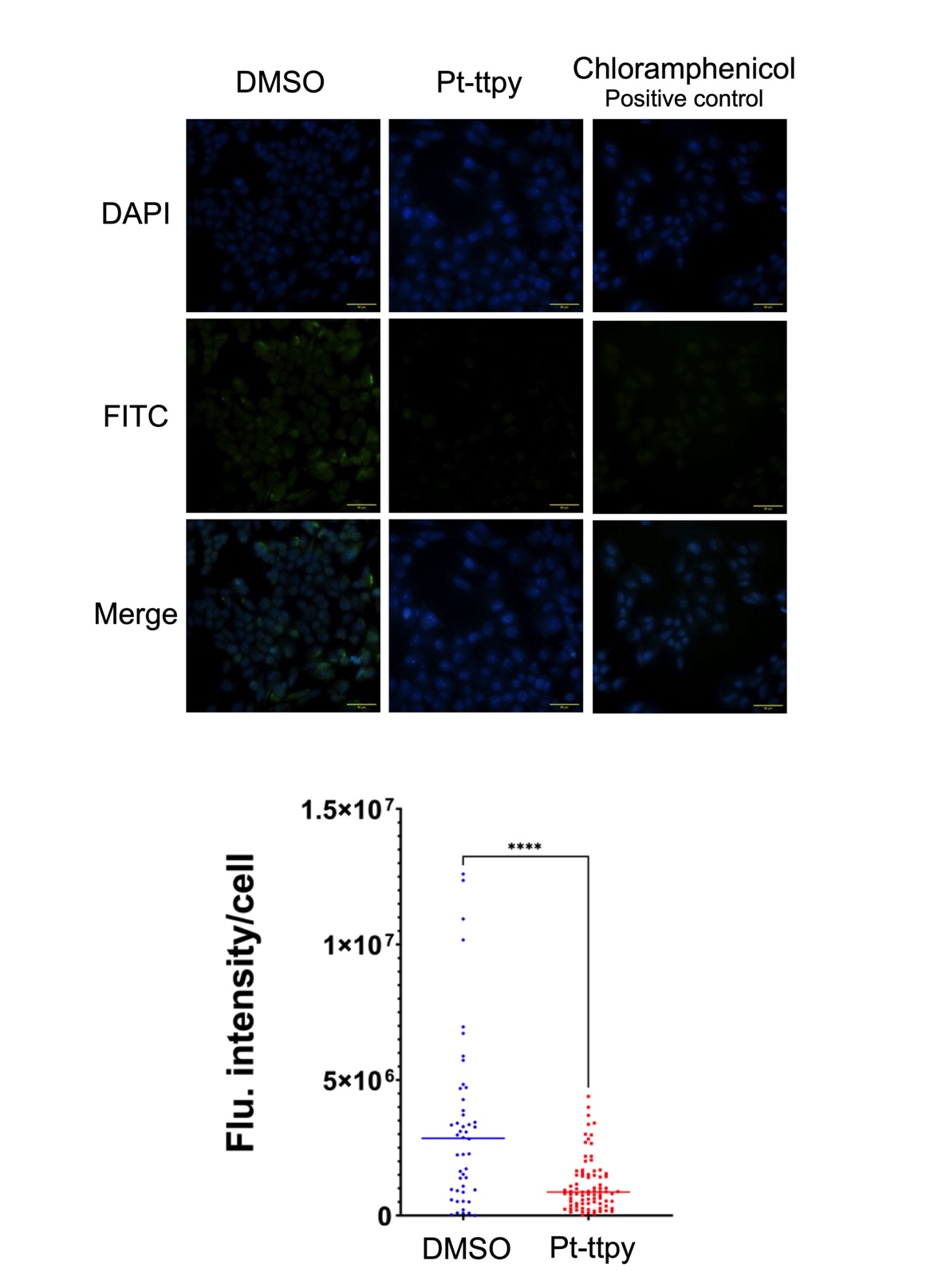


**Fig. S12.** UP: represented figures of fluorescent imaging of the mitochondria under Pt-ttpy and DMSO treatments in Hela cells. A Positive control of blocking the synthesis of mitochondrial proteins by chloramphenicol was also presented. Down: Single-cell quantification showed Pt-ttpy significantly inhibited mitochondrial translation, DMSO group (>50), Pt-ttpy group (n>50). Data represents two independent experiments. P values were calculated toward the DMSO group: *P < 0.05, **P < 0.01, ****P < 0.0001, unpaired t-Student test.


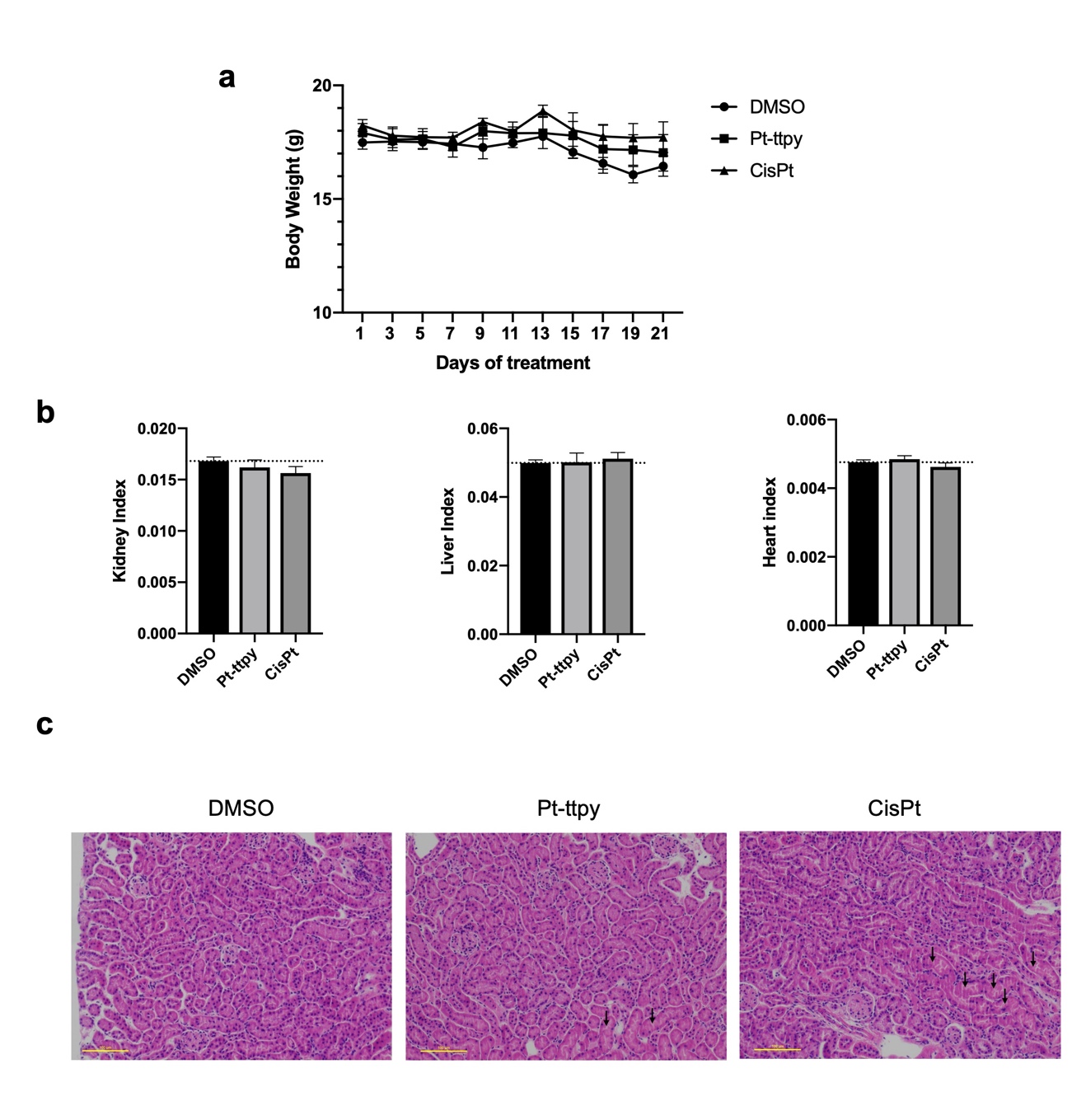


**Fig. S13**. Cisplatin exhibited more toxicity to kidney, as compared with Pt-ttpy. (a) Body weight curve of mice under the treatments of DMSO, Pt-ttpy and CisPt. (b) Index of the major tissues, including kidney, liver, and heart, post indicated treatments. Data is represented as mean ± SEM. (c) The Pt-ttpy and cisplatin groups exhibited turbid staining, edema (↓), indistinct intercellular boundaries, and uneven nuclear staining in the epithelial cells of renal proximal tubules, with the cisplatin group showing a more pronounced manifestation of these characteristics. The images are represented for three mice in each group.


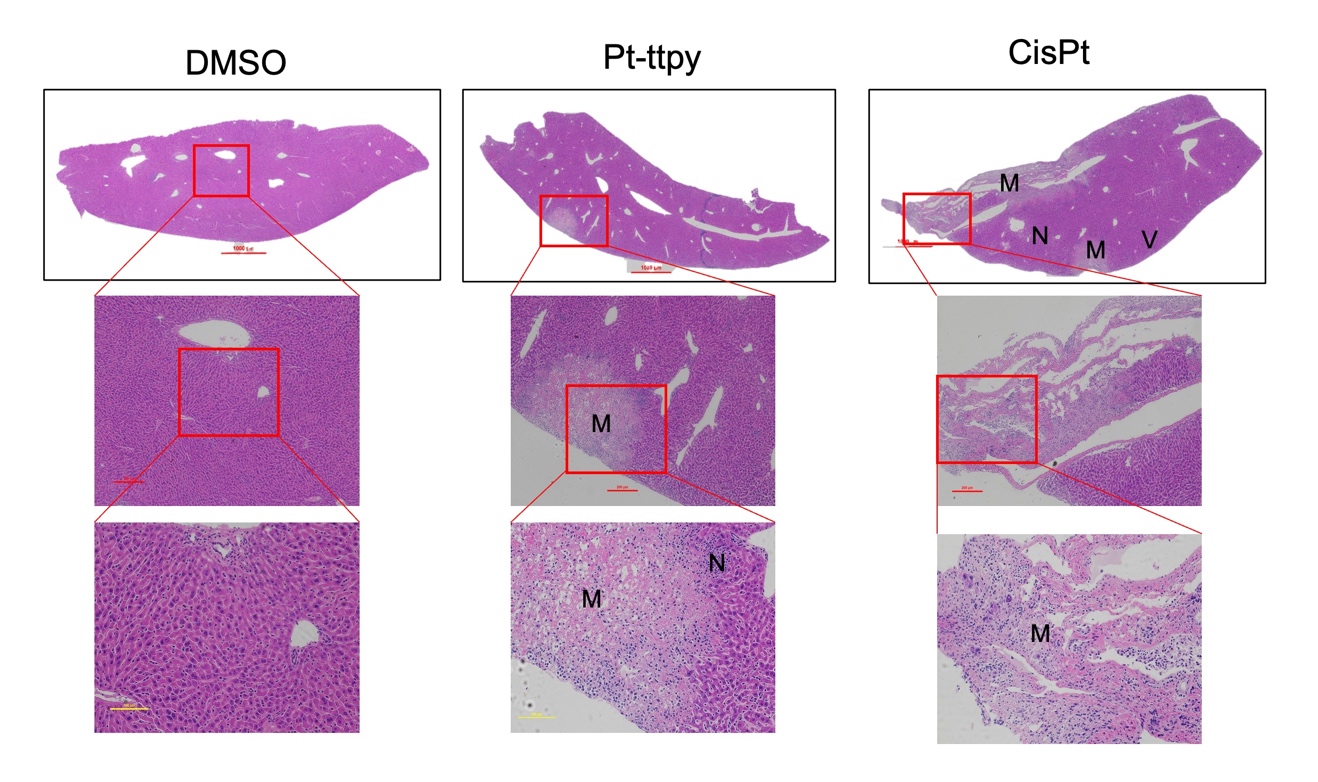


**Fig. S14.** Cisplatin induced more toxicity to liver as compared with Pt-ttpy. Up: full scan of liver post indicated treatments. Down: Pt-ttpy induced neutrophil infiltration (N) in some areas without hepatocyte apoptosis, and cisplatin induced massive inflammation (I) around portal vein and blood vessels with enlarged sinusoidal space and vascular congestion (V). The images are represented for three mice in each group.

Table S1. A list of Primers for PCR

| **Gene** | **Fw sequence (5’→3’)** | **Rv sequence (5’→3’)** |
| --- | --- | --- |
| Actin | TCACCCACACTGTGCCCATCTACGA | CAGCGGAACCGCTCATTGCCAATGG |
| 12S | TAGCCCTAAACCTCAACAGT | TGCGCTTACTTTGTAGCCTTCAT |
| tRNA-Leu(UUR) | CACCCAAGAACAGGGTTTGT | TGGCCATGGGTATGTTGTTA |
| ND4 | TCTGTGCTAGTAACCACGTTC | AAAACCCGGTAATGATGTCG |
| COX III | CCACTCCTAAACACATCCGTA | GCCAATAATGACGTGAAGTCC |
| mt-deletion | TTCCTCATCACCCAACTAAAAA | TTCGATGATGTGGTCTTTGG |
| COX I | AATAGGAGCTGTATTTGCCAT | AGAAAGTTAGATTTACGCCGAT |
| Cytb | TTATTGACTCCTAGCCGCAGA | TAGTACGGATGCTACTTGTCCA |
| ND6 | ATATACTACAGCGATGGCTA | AATCCTACCTCCATCGCTA |
| AURKAIP1 | GCAAAAACGTGCTGAAGATCCGC | GCCTCAGGTCTTTCTCGAACTTG |
| DHX30 | GAGCACTACCTAGAGGACATCC | CGATGTGCAGAACCAGATCAGTC |
| ERAL1 | CTGGACCACTTCCTCGGATTCT | TCAGGGTGATGGACCAAGAGGA |
| GUF1 | GATTCCAGTGTGACCGTTCATCG | TTCTTGCTCCAGTCGCTGGTTG |
| MPV17L2 | AGGGTCAGACAGTGGGTGAGAG | GCCGTTGATGTAGGTGACTCGA |
| MRM2 | GTGAACGAGAGGCACCAGATTC | CAAGCACGAAGCCAACAGGAGA |
| MRM3 | AAGAGTCTGGGCTTCGCTACGA | AGAGCGTCTGAAATGAGCCTGC |
| MRPL12 | CTACATCCAAGGCATCAACCTCG | GATCTTCTCCGCCTCAGCTTTG |
| MRPL15 | AGGTGTGACCATCCAGCCACTT | GCAGCAATAGCTAGTTCTGAAGC |
| MRPL18 | TGGCACAGAGATGCTTAGAGGC | CACACCACCTTCTGTCATGGCA |
| MRPL20 | TACAGCTGCTAGCCAGGAACATG | CAGATCCGCTAGGACTTTCCTG |
| MRPL32 | GGTGTAGGAGAAGAAATCCGCAG | GCACACCTTTTCATAGCAGTAGG |
| MRPL36 | AGCAGTGCGCTCACTTCTCTCA | CCTCTTCACCAGGTAACAGTCC |
| MRPL37 | ACAGACCTGGACTGTAACGAGG | CTCTGGCTTGAAACCAACTGGG |
| MRPL38 | TGTTCCACGGTGCCACCTTTGT | CTCTTCTGCCTCATAGGTCACC |
| MRPL41 | TCAAGCCCTACGTGAGCTACCT | AGGTTGTCAGGGTCGAAGGTAC |
| MRPL44 | TCCAGAAGGAGTTAGAGCGGCA | GGAAAAGTTTTCCTGTAACCGATG |
| MRPL46 | CCTTCGAGGAACAGCTGAACGA | CGAGGTTACTCTCTGTCCGCAT |
| MRPL49 | CAGTTTGTGGAGCGCCTGTTAC | TTGTGCATCCGAGAGCGTCGTA |
| MRPL52 | GAGCTCCCAGACTGGTCATATG | CAGCGTCCATTTCCTGTGACAG |
| MRPS11 | CCTTTGCTTCCTGTGGCACAGA | GCCTTTCACCACAACTCGGATG |
| MRPS18C | CAGGTATCCAGCAATGAGGACC | GCATCCAGTAAATGGAGAAACAAAC |
| MRPS30 | GGCTTTTGAGACAAAACTGTGCTG | GGTCGAGTAACATCTGCTTCACT |
| MRPS5 | GAAGAGTGTTCTCGGCAATGGC | TACTGCTGGCTCATCAGGTGAC |
| MRPL43 | GAGGAGAGCATCCACTGCAA | CCTGGATGCTAGGGTTGTCG |
| MRPS24 | TGAGGCAGTTGTCTCCACAC | ACACAACCTTTGAGGGCACA |
| MRPL27 | CGGACAGCCGTTACATCCTT | GCCTGATGACTTTCCACCGA |
| MRPS26 | GCTGGCCAAATCCAAGATCG | CTGGTAACGCTCCATCAGCA |
| MRPL34 | CACTGCGGATATGGCTGTCT | TGATACTCATTCCCGCGAGC |
| MRPL11 | AGGCGTTTCCATCAACCAGT | CTGCCACCTCTTTCCCTGTT |
| MPV17L2① | CAGGCAGTAGTTTGCGACTC | TGCTGGGATGATGATAGGCG |
| MPV17L2② | GCCACAGATCACATCCCTTCT | TGGTTGCCCTACGTATTTCCA |
| MPV17L2③ | CGGGACGTAACGCAATTCTT | TGACCAATAAAAGGCAGGCG |
| MPV17L2④ | GTTCCTTGGTTCCTGAGGGC | AGCGTGTTAGTGACGAGCAG |
| MPV17L2⑤ | ACGCTGCTTAGTCCTTCACC | AGTCGGGTGTCGGGATCAAA |
| MRPS18C① | CTTAACTGCTGCGTGCACAA | TCGTTACCACTGTTAATCGGCA |
| MRPS18C② | CAAGTCCTCCGTACCTGGTC | GGACCTCCTACGTCACATGC |
| MRPS18C③ | ACCGGAAGCACGCATAAACT | GCAGGTCACGTGAGGCTTAT |
| MRPS18C④ | TTGCTGTTTGCGGTGGTCTA | GCGCTACAATGGAGCATAGGA |
| MRPS18C⑤ | AAGGGACATTTCCAGAGTCGC | AAAGTAACAGACCCGTCCCAG |

**SI References**

1. O. Rothfuss, T. Gasser, N. Patenge, Analysis of differential DNA damage in the mitochondrial genome employing a semi-long run real-time PCR approach. *Nucleic Acids Res* **38**, e24 (2010).

2. J. T. Robinson, H. Thorvaldsdottir, D. Turner, J. P. Mesirov, igv.js: an embeddable JavaScript implementation of the Integrative Genomics Viewer (IGV). *Bioinformatics* **39** (2023).
